# A molecular signature of dormancy in CD34^+^CD38^-^ acute myeloid leukaemia cells

**DOI:** 10.18632/oncotarget.22808

**Published:** 2017-11-30

**Authors:** Mazin Gh. Al-Asadi, Grace Brindle, Marcos Castellanos, Sean T. May, Ken I. Mills, Nigel H. Russell, Claire H. Seedhouse, Monica Pallis

**Affiliations:** ^1^ University of Nottingham, School of Medicine, Academic Haematology, Nottingham, UK; ^2^ University of Basrah, College of Medicine, Basrah, Iraq; ^3^ University of Nottingham, School of Biosciences, Nottingham, UK; ^4^ Centre for Cancer Research and Cell Biology, Queen’s University Belfast, Belfast, UK; ^5^ Clinical Haematology, Nottingham University Hospitals, Nottingham, UK

**Keywords:** AML, dormancy, gene expression profiling

## Abstract

Dormant leukaemia initiating cells in the bone marrow niche are a crucial therapeutic target for total eradication of acute myeloid leukaemia. To study this cellular subset we created and validated an *in vitro* model employing the cell line TF-1a, treated with Transforming Growth Factor β1 (TGFβ1) and a mammalian target of rapamycin inhibitor. The treated cells showed decreases in total RNA, Ki-67 and CD71, increased aldehyde dehydrogenase activity, forkhead box 03A (FOX03A) nuclear translocation and growth inhibition, with no evidence of apoptosis or differentiation. Using human genome gene expression profiling we identified a signature enriched for genes involved in adhesion, stemness/inhibition of differentiation and tumour suppression as well as canonical cell cycle regulation. The most upregulated gene was the osteopontin-coding gene SPP1. Dormant cells also demonstrated significantly upregulated beta 3 integrin (ITGB3) and CD44, as well as increased adhesion to their ligands vitronectin and hyaluronic acid as well as to bone marrow stromal cells. Immunocytochemistry of bone marrow biopsies of AML patients confirmed the positive expression of osteopontin in blasts near the para-trabecular bone marrow, whereas osteopontin was rarely detected in mononuclear cell isolates. Unsupervised hierarchical clustering of the dormancy gene signature in primary acute myeloid leukaemia samples from the Cancer Genome Atlas identified a cluster enriched for dormancy genes associated with poor overall survival.

## INTRODUCTION

Acute myeloid leukaemia (AML) is characterised by a high disease relapse rate despite initial induction of remission. The leukaemia-initiating cells (LIC) that survive chemotherapy are likely to be enriched for a dormant subpopulation within the bone marrow niche [1]. Dormancy is a non-proliferative state where cells stay outside the cell cycle but retain their proliferative potential, in contrast to the terminally non-proliferative differentiated cell. The study and characterization of dormant LICs could pave the way to their targeting and eradication, but the simple paradox that these rare dormant cells cannot be expanded for research without breaking their dormancy suggests that efforts need to be directed to establish experimental models of induced dormancy.

The crosstalk between LICs and the bone marrow (BM) niche in which they reside has been implicated in dormancy regulation. Leukaemia cells which are responsible for relapsed AML, similar to normal haematopoietic stem cells (HSCs), reside in a low perfusion microenvironment in the endosteal region of the bone marrow [2–4]. The scarcity of nutrients in the poorly-perfused niche is likely to contribute to leukaemia cell dormancy and resistance to chemotherapy. MTOR inhibition by rapamycin can be used to mimic shortage of nutrients [5, 6], and has been employed for the *in vitro* maintenance of haematopoietic stem cells [7]. Furthermore, it has been reported that mTOR-inhibited leukaemia cell lines [8] and prostatic cancer cells [9] showed features of dormancy and resistance to chemotherapy. TGFβ1 is strongly involved in the regulation of dormancy in normal undifferentiated HSC [10]. The addition of mTOR inhibition to TGFβ1 was reported to potentiate the inhibitory effect of TGFβ1 in transformed cells [11].

The current study aimed to exploit the above two key BM LIC niche characteristics – i.e. the abundance of TGFβ1 and shortage of nutrients, to establish an *in vitro* model that enables molecular characterization of dormant AML cells. Some AML clones are dependent on aberrant activation of the mTOR pathway for survival and hence sensitive to clinical mTOR inhibitors, but other clones are resistant [12], and in this study we characterise a cell line (TF-1a) which remained viable in the presence of rapamycin and in which we were able to exploit the dormancy-inducing physiological role of mTOR inhibition [13]. This work is a step towards the ultimate goal of finding molecular targets that might help to eradicate dormant LICs and hence prevent relapse.

## RESULTS

### TGFβ1 and mTOR pathway inhibition significantly impede TF-1a cell proliferation and induce features of dormancy and stemness without affecting cell viability or inducing cell differentiation

**Figure 1:**
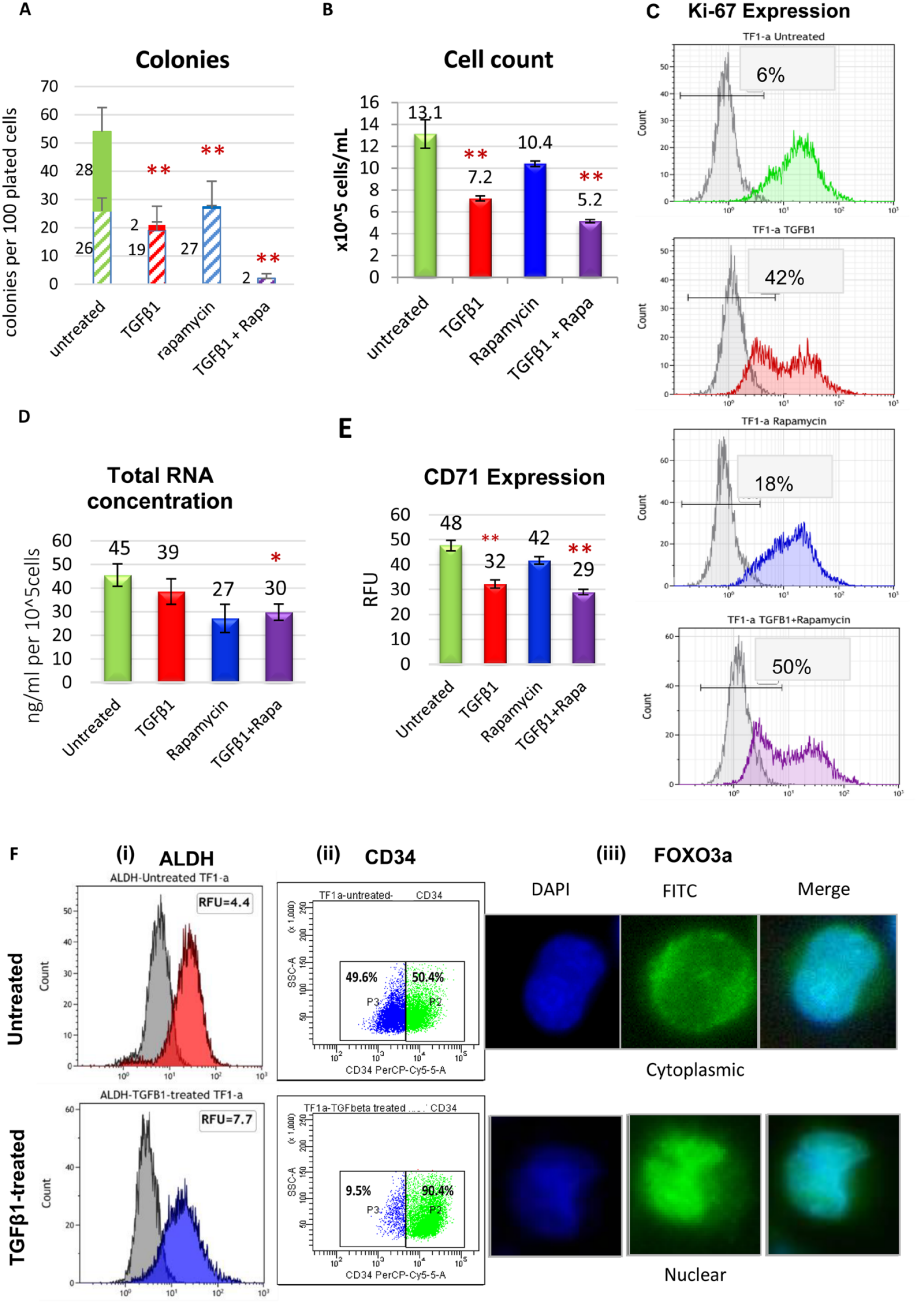
Dormancy induction in TF-1a cells **(A)** Clonogenic assays were carried out in a methylcellulose-based medium (H4100 from Stem cell Technologies) using standard procedures, with TF-1a cells seeded at 200 cells per 100 μl in triplicate in 96-well plates with 4ng/ml TGFβ1 and/or 100nM rapamycin. Large colonies (solid upper bars, >50 cells) and smaller colonies (striped lower bars, 20-50 cells) were counted after 6 days. **(B)** Inhibition of growth in suspension culture. Cells were seeded at a concentration of 2x10^5 cells/ml and incubated for 72 hrs with 4ng/ml TGFβ1 and/or 100nM rapamycin before counting. **(C)** Cells were cultured for 3 days as in B: flow cytometric histograms of Ki-67 expression. Isotype control (grey histograms) are used as a reference to determine the percentage of Ki-67 negative cells. **(D)** Cells were cultured for 3 days as in B. Total RNA content was measured using a nanodrop. **(E)** Cells were cultured for 3 days as in B: surface CD71 expression measured by flow cytometry. **(F)** Modulation of stemness markers by TGFβ1: ALDH (i), CD34 (ii) and FOXO3a (iii). In A, B, D and E, the columns represent mean +/- SD of three independent experiments (^*^: *p* < 0.05; ^**^: *p* < 0.01). In C and F, the figures are representative examples of 3 biological replicates.

TF-1a cells were cultured with 4ng/ml TGFβ1 and/or 100nM rapamycin. Clonogenic growth was inhibited by TGFβ1 or rapamycin individually, and the combination of the two agents blocked the formation of colonies (>95% inhibition, Figure 1A). Growth inhibition in suspension culture (Figure 1B) was accompanied by features of dormancy including an increase in the proportion of Ki-67 negative cells (p < 0.01) (Figure 1C) and a decrease in RNA, characteristic of dormant cells due to their low metabolic activity (Figure 1D). In addition, a significant decrease in the transferrin receptor and proliferation marker CD71 was evident (Figure 1E). TGFβ1+rapamycin treatment showed no effect on the viability of TF-1a cells as determined by annexinV flow cytometry assay (Supplementary Figure 1), excluding cellular death as a possible explanation for the growth inhibition. The cellular potential to proliferate decreases in the haematopoietic cell hierarchy as cells differentiate. However, following conditioning with TGFβ1 and/or rapamycin, TF-1a cells maintained a primitive morphology with no signs of differentiation (Supplementary Figure 2). TGFβ1 is reported to upregulate the stem-like properties of HSC [14] and LIC [15], and AML cells with intermediate ALDH activity are reported to be highly represented in minimal residual disease AML samples [16]. TGFβ1 upregulated ALDH activity and surface CD34 expression in TF-1a cells. Of note, although CD34 expression ranges from negative to positive in untreated TF-1a cells the treated cells became >90% CD34+ (Figure 1F and Supplementary Figure 3). Relocation of FOXO3a from cytoplasm to nucleus, suggestive of activation, was also seen (Figure 1F). Growth-inhibitory responses of KG-1a, Kasumi-3 and M0-91 cells to TGFβ1 were also measured as well as their CD34 and CD38 status (Supplementary Figure 3). The growth-inhibitory response to TGFβ1 noted in TF-1a cells compared to KG-1a and Kasumi-3 cells and the pronounced loss of Ki-67 in TF-1a after TGFβ1 treatment compared to Kasumi-3 and M0-91 cells supported its selection to model and characterize AML cell dormancy in the study.

### Molecular signature of TF-1a cells induced to dormancy

**Figure 2:**
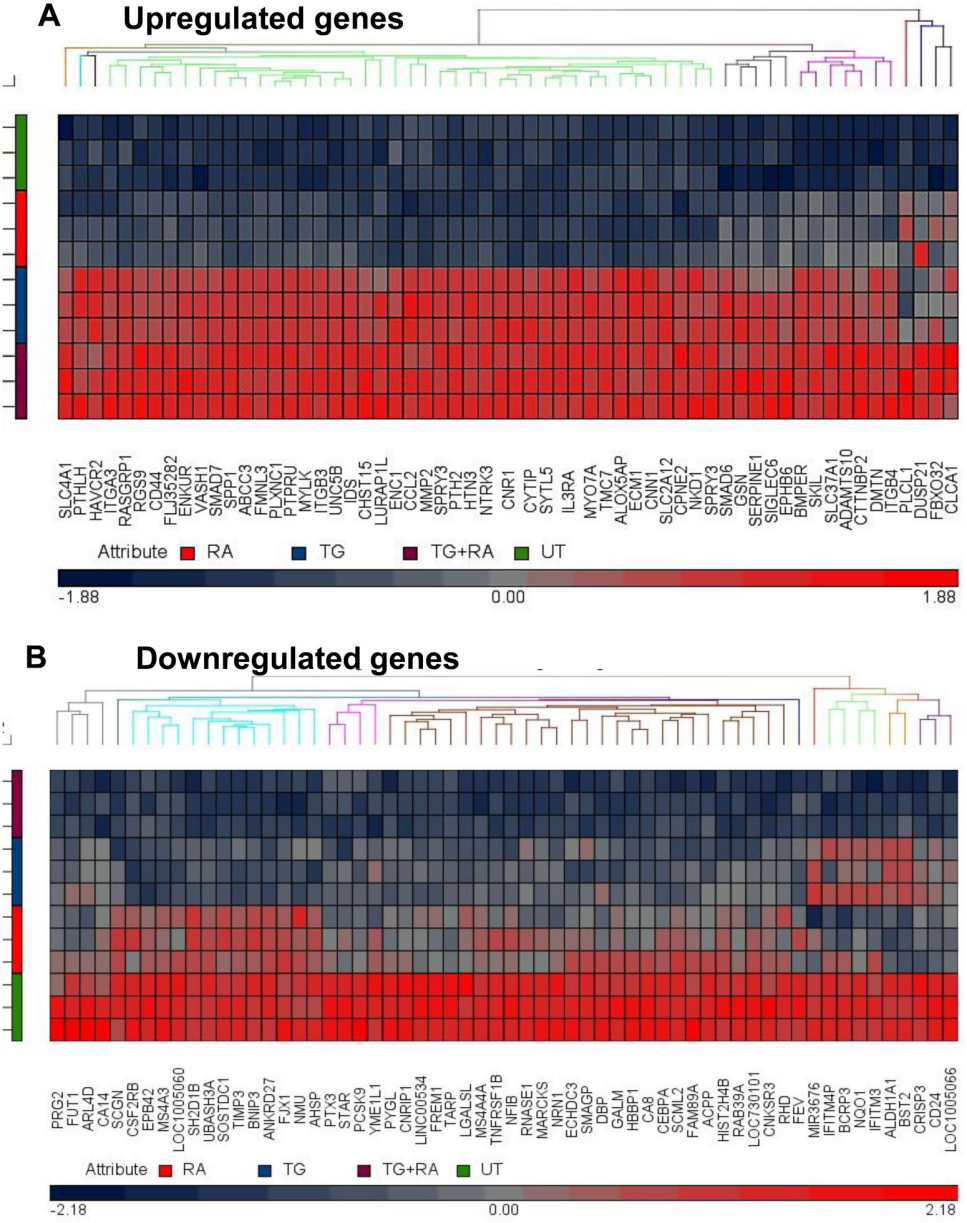
Heat maps of differentially regulated genes in dormant TF-1a cells TF-1a cells were cultured for 3 days with 4ng/ml TGFβ1 and/or 100nM rapamycin prior to gene expression profiling. The heat maps illustrate **(A)** The top 60 of the 240 most significantly upregulated genes and **(B)** the top 60 of the 136 most downregulated genes. UT: untreated; RA: rapamycin; TG: TGFβ1.

Dormancy-induced TF-1a cells were subjected to molecular characterization by microarray analysis. Restricting the dormancy signature to genes upregulated or downregulated by at least 2 fold with false discovery rate (FDR)-adjusted p value <0.05, we found 240 upregulated genes and 136 downregulated genes in dormant TF-1a cells compared to their untreated (cycling) counterparts (Figure 2 and Supplementary Figure 4). A full list of the differentially regulated genes can be found in Supplementary Tables 1 and 2) and a copy of the complete results have been deposited in the gene expression omnibus database, (http://www.ncbi.nlm.nih.gov/geo/), reference GSE102483. As is clear from Figure 2, TGFβ1 alone contributes much more strongly to gene upregulation than rapamycin, but the disparity is not as strong in downregulated genes.

**Figure 3:**
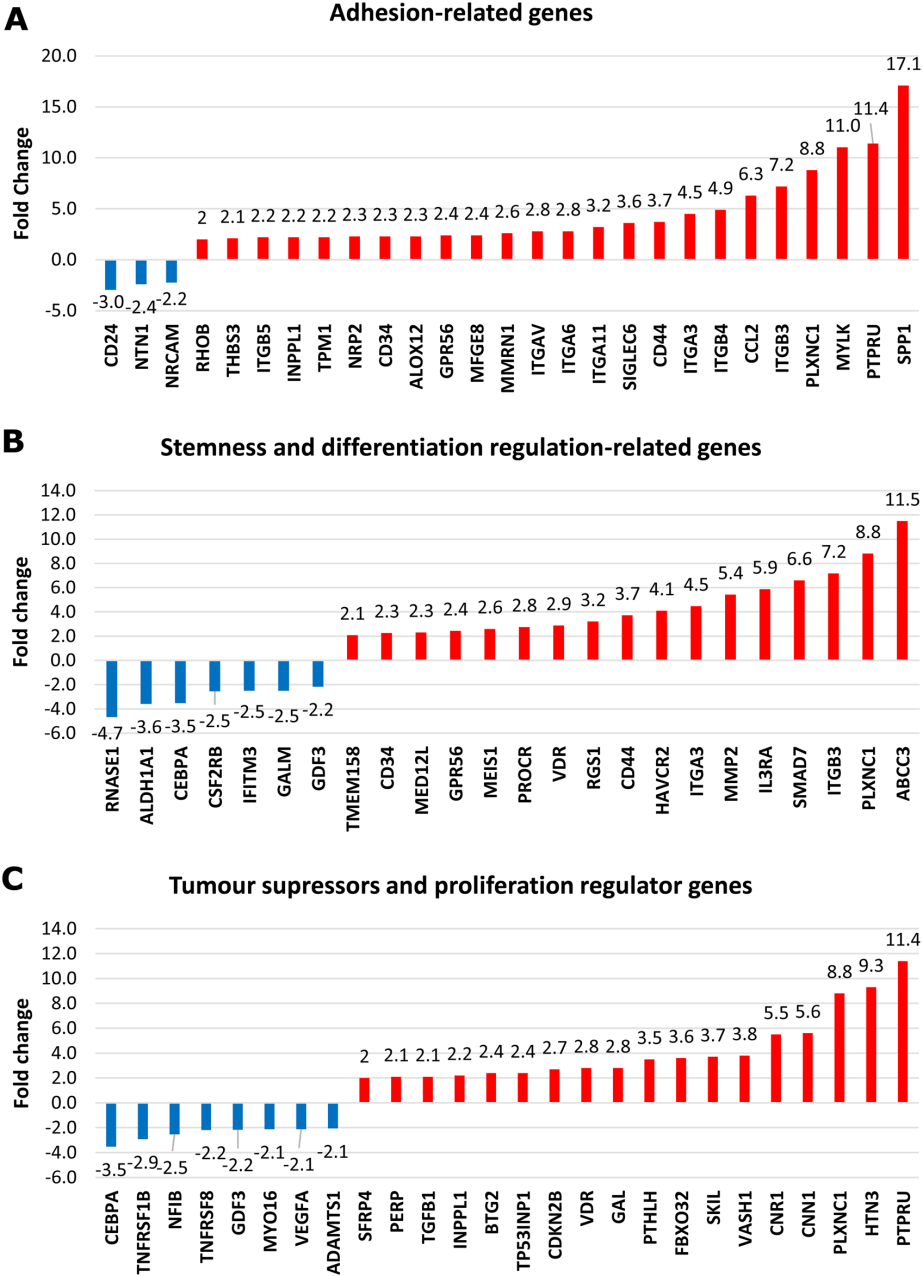
Biologically related groups of genes in dormant TF-1a cells Mean values for fold upregulation (red bars) or downregulation (blue bars).

Among the 240 differentially upregulated and 136 downregulated genes in dormant TF-1a cells, we identified canonical targets of TGFB1 and of mTOR inhibition (Supplementary Table 3). We found three biologically relevant groups of genes highly represented in the dormancy signature. These were adhesion-related, tumour suppressor and stemness / inhibition of differentiation-related genes (Figure 3). The categories overlap: for example, amongst the adhesion related genes, ITGB3 [17] and CD44 [18] are also essential for the maintenance of leukaemia initiating cells.

### Integrin and cell adhesion signalling pathways dominate the biological processes enriched in the molecular signature of dormant AML cells

**Figure 4:**
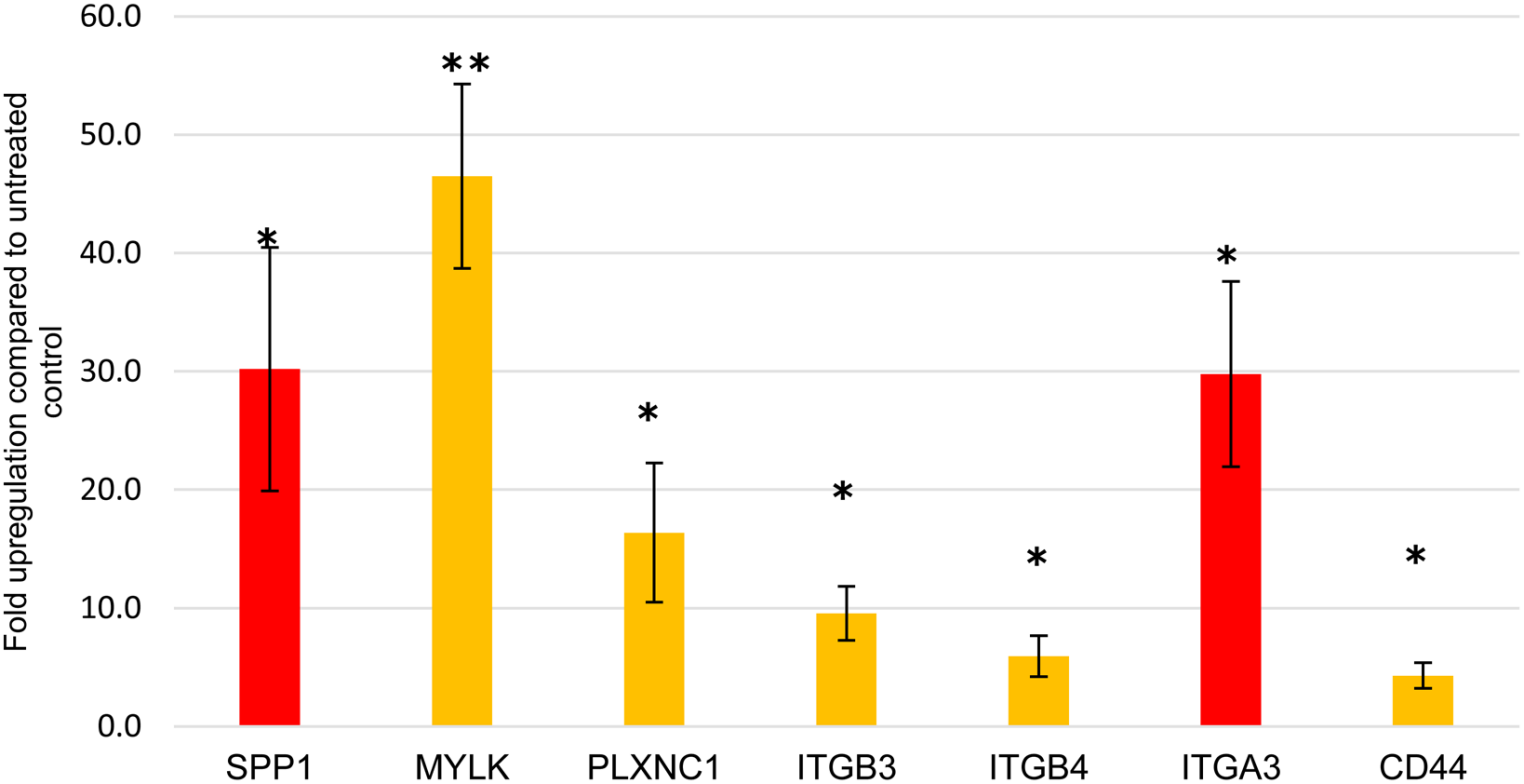
qPCR validation Expression of the 7 top significantly upregulated adhesion-related genes in dormant TF-1a cells. SPP1 and ITGA3 genes are highlighted in red as their baseline expression was negligible, in contrast to the other genes where the increase was indicative of upregulation. Mean +/- SD of three independent experiments. ^*^ p value<0.05, ^**^ p value<0.01.

To group the detected genes based on their gene ontology (GO) categories into relevant biological processes, gene enrichment analysis was performed (in Partek) using the Kyoto Encyclopaedia of Genes and Genomes (KEGG) database. Information was available on 195 of the 240 upregulated genes, and these mapped to 56 distinct biologically relevant groups (Supplementary Table 4). Among these, the integrin-mediated and cell adhesion signalling pathways showed the highest enrichment scores; 14.43 and 13.41 respectively (p<0.001 for both). Confirmation of adhesion-related gene upregulation was obtained by quantitative PCR (Figure 4). Information on 100/136 downregulated genes was available and these were mapped to 46 significantly enriched distinct biologically relevant groups (Supplementary Table 5). Downregulated genes were dominated by alterations in metabolic pathways.

### Osteopontin is expressed in AML bone marrow and its expression is stimulated by TGFβ1 in primary samples

**Figure 5:**
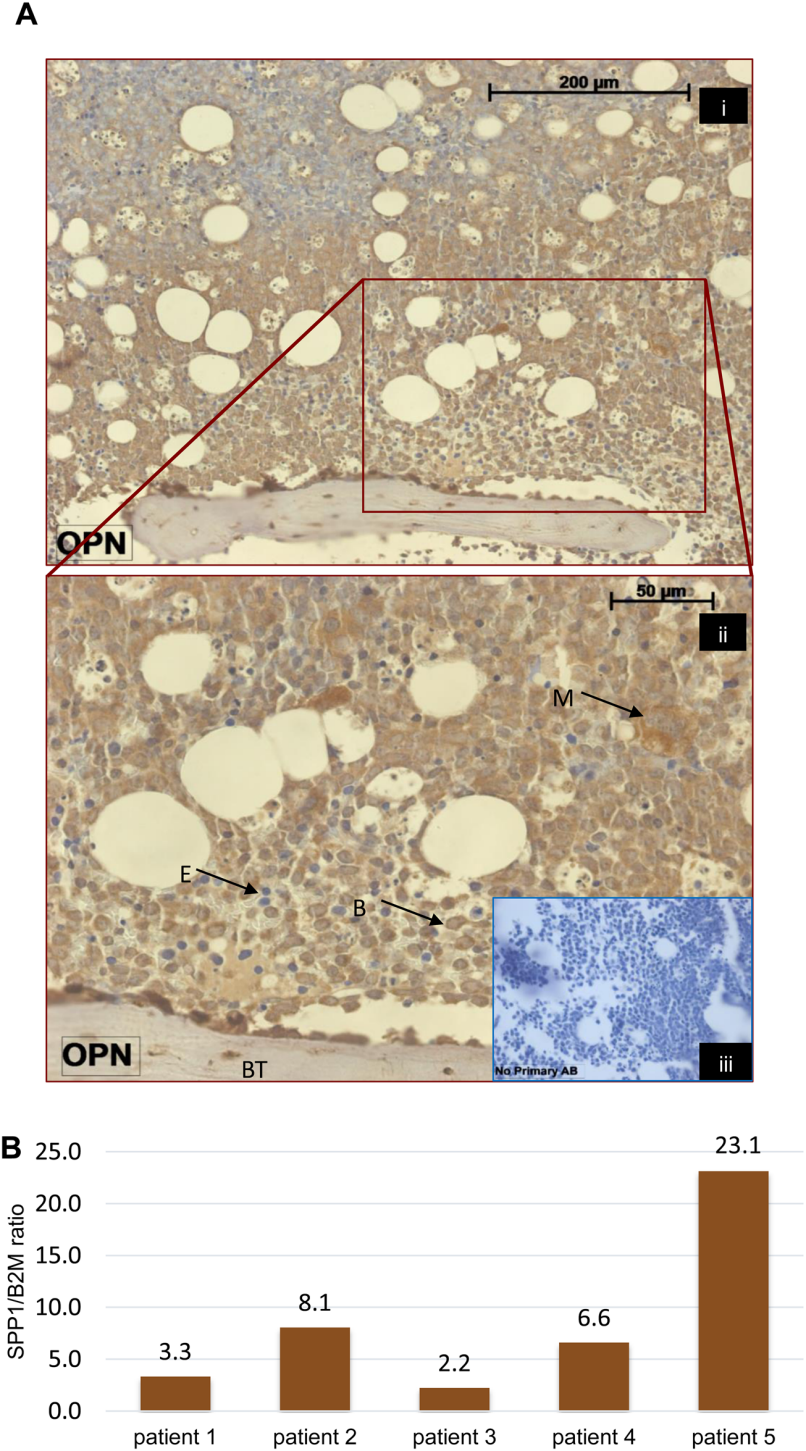
Osteopontin expression in AML patients **(A)** Osteopontin expression in a bone marrow biopsy from an AML patient. Picture (ii) shows magnification of the rectangular area from picture (i) to illustrate the cytoplasmic expression in leukemic blasts. B, blast. BT, bone trabecula. E, erythroid cell. M, megakaryocyte. (iii) No primary antibody control. **(B)** SPP1 expression in primary AML cells in response to 3 day *in vitro* culture with 4 ng/ml TGFβ1. SPP1 message was measured by qPCR in 5 samples. Bars represent fold upregulation of SPP1:B2M ratio compared to untreated controls.

SPP1, the gene encoding osteopontin, showed the highest fold upregulation in our model. However levels (even though significantly upregulated) were low and attempts at protein detection in the model were unreliable. A meta-analysis of several studies indicates that serum osteopontin (OPN) is a marker of poor survival and a potential therapeutic target in AML patients [19]. We therefore looked for the presence of osteopontin in primary AML patient material. Osteopontin protein was detected in the blast cell cytoplasm of both presentation and post-treatment BM biopsies from 7/7 patients (patient details in Supplementary Table 6). 2 of the biopsies showed stronger OPN expression in the cytoplasm of blasts adjacent to the bone trabeculae in comparison to those further from this area of the BM (Figure 5A). The other 5 cases showed a similar pattern of OPN expression regardless the anatomical localization of the leukaemia cells (e.g. Supplementary Figure 5). We also measured SPP1 message in 13 primary AML mononuclear cell isolates. In 10/13, SPP1 expression was undetectable (Supplementary Figure 6), strongly suggesting the necessity for exogenous factors present in the bone marrow microenvironment such as TGFβ1 to induce expression. Indeed SPP1 expression was induced by *in vitro* culture with TGFβ1 in 5/5 samples (Figure 5B).

### Protein and functional validation of adhesion proteins in AML cells

**Figure 6:**
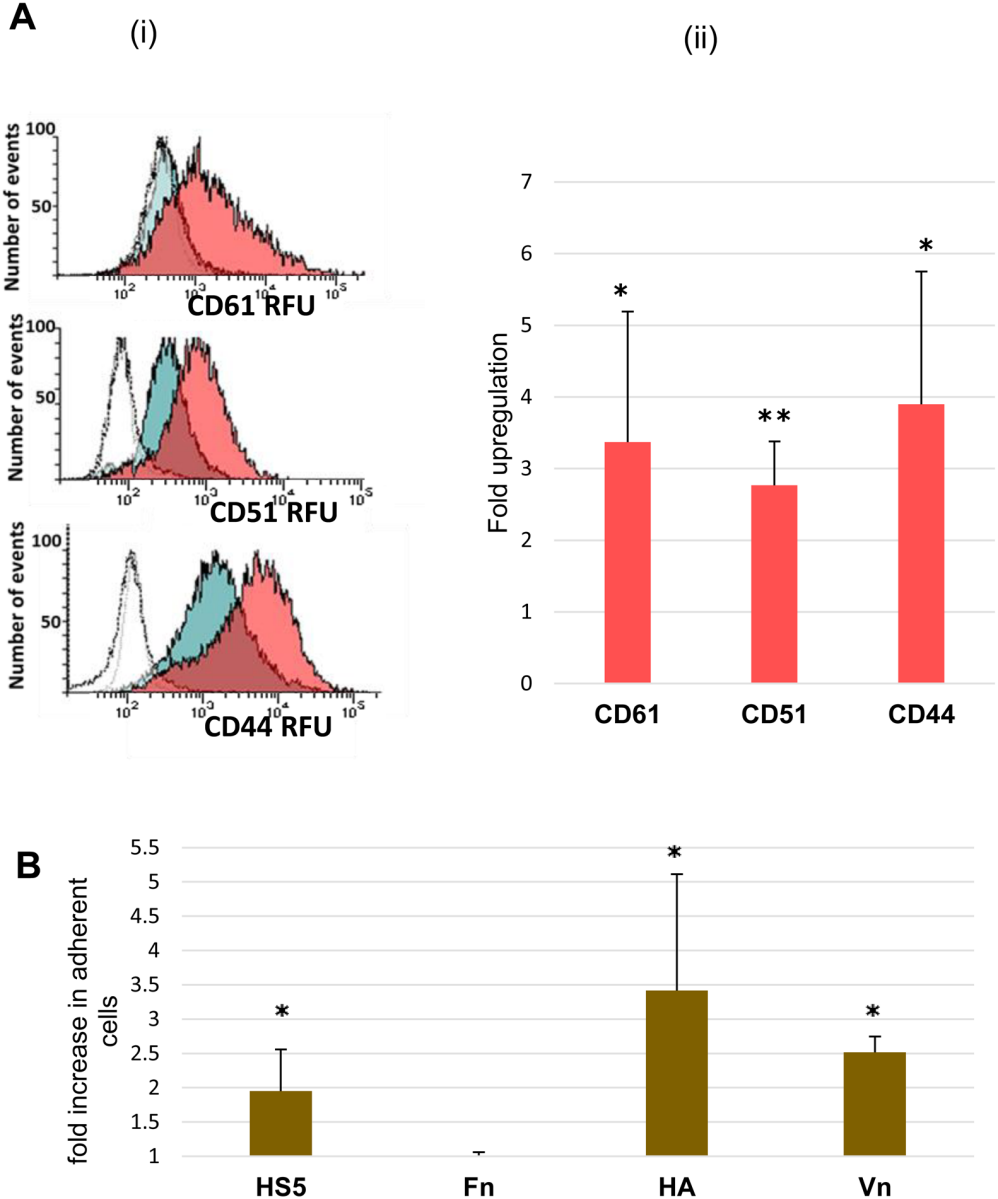
Adhesion molecule expression and *in vitro* adhesion of dormant TF-1a cells **(A)** (i) Flow cytometric histograms illustrating CD61, CD51 and CD44 RFU (relative fluorescence units) in cycling TF-1a (blue-filled histogram), and dormant cells (pink-filled histogram). Unfilled histograms represent isotype control fluorescence (in the case of CD61, the blue-filled histogram almost completely overlaps the isotype control). (ii) Summary plot (Mean + SD) for 3-6 independent assays. **(B)** The ratio of treated to untreated cells adherent to HS-5 stromal cells and to plates coated with fibronectin (Fn), hyaluronic acid (HA) or vitronectin (Vn), (Mean + SD for 3-5 independent assays). ^*^= p value<0.05, ^**^= p value<0.01.

As genes involved in integrin-mediated signalling and adhesion were markedly enriched in the signature of dormant AML cells, we further characterised the most upregulated integrin – integrin beta 3 (ITGB3, CD61) and its binding partner integrin alpha v (ITGAV, CD51). We found upregulation of CD61 (p=0.01) and CD51 protein expression (p=0.005) in the dormancy-enriched cells (Figure 6A). CD44, a gene found in the dormancy expression profile which interacts with ITGB3 and osteopontin to mediate adhesion and signalling (reviewed in [20] and [21]), was also upregulated at the protein level (p=0.01).

We also examined the adhesive properties of the dormancy-enriched TF-1a cells (Figure 6B), which demonstrated significantly enhanced adhesion to the stromal cell line HS-5 (p=0.03). Dormant cells were also more adherent than cycling cells to immobilised vitronectin (fold increase 2.5; p=0.02) and hyaluronic acid (fold increase = 2.4; p=0.02), (ligands of integrin B3 and CD44 respectively). As expected from the literature on this topic, fibronectin elicited strong adhesion [22], but this was not increased in dormant cells.

### Molecular signatures of dormancy in patient samples

**Figure 7:**
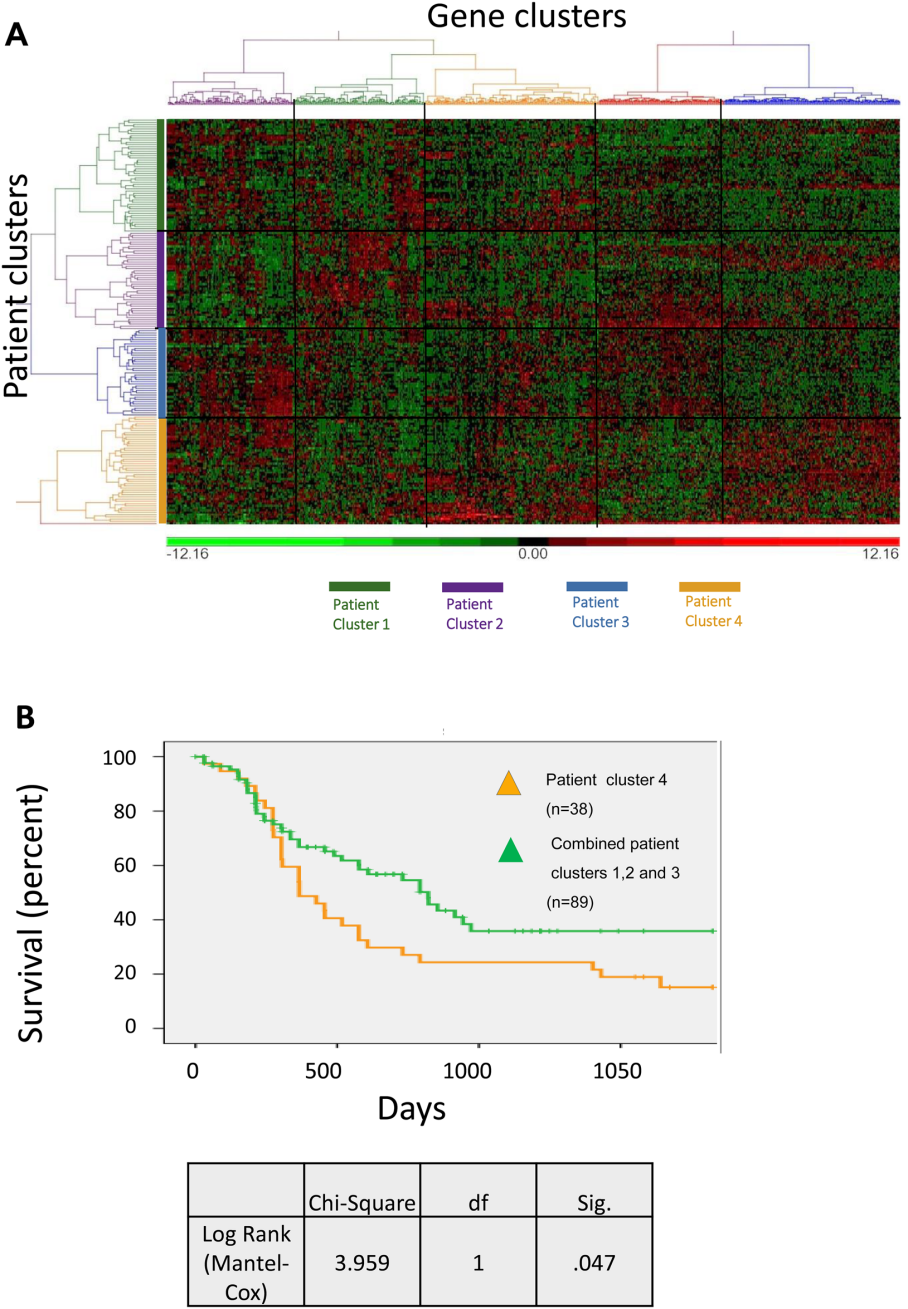
Dormancy signatures in TCGA patient samples Analysis of samples from TCGA LAML database, which included 778 probes for 341/376 genes (240 upregulated and 136 downregulated) of the dormant TF-1a cell signature**. (A)** Un-supervised hierarchical clustering of the probes in 149 AML patient samples**. (B)** Survival curve of AML patients from Patient Cluster 4 (gold line) compared to the combined Patient Clusters 1, 2 and 3 (green line).

Publically available data from the Cancer Genome Atlas (TCGA) [23, 24] on 149 cases of AML (excluding patients with a t(15;17) translocation, but comprising all other patients with documented outcome data) was studied to determine whether there is a particular subset of AML patients with a strong intrinsic dormancy signature. Using unsupervised hierarchical clustering, we selected the 240 upregulated and 136 downregulated genes identified in our model, from which we mapped five clusters of genes and four clusters of patients (Figure 7A). A fifth patient cluster comprising a single case was excluded from the analysis. Using Fisher’s exact test we determined significant enrichment for adhesion in gene cluster 5 (P=0.02), for stemness in gene cluster 3 (P=0.033) and for tumour suppressors in both gene cluster 4 (P=0.009) and cluster 5 (P=0.03) (Supplementary Table 7). The four patient clusters were found to have significantly different proportions of highly expressed (>median) dormancy genes (1<2<3<4). The overall survival of intensively treated patients (n=127) was significantly worse in the cluster with the highest number of dormancy genes upregulated, i.e. patient cluster 4 (P=0.047, Figure 7B). This patient cluster contained a low proportion of FLT3 mutations (P=0.051). It also contained all 6 FAB M6 and M7 cases, but was not enriched for a particular cytogenetic risk group, age or mutation in NPM1 (Supplementary Table 8). Intensively treated patients were dichotomised for one year survival. After excluding patients still alive but censored at less than one year, 106 patients remained in the dataset (61 alive, 45 dead). In this group the ten most differentially over-expressed genes in patient cluster 4 were entered as potential predictors into multivariate (backward conditional) analysis. (Where there was more than one probe per gene, only genes overexpressed in cluster 4 with at least 3 out of 4 probes were evaluated.) Expression levels of ITGB3, IL6ST and PTH2 were found to be significant prognostic factors of 1 year survival. Cytogenetic risk group, age and the presence of FLT3-ITD and NPM1 mutations were entered (Supplementary Table 9), but were not found in the final model.

## DISCUSSION

The establishment of an *in vitro* model of dormancy in AML cells was achieved by exploiting two prominent features of the dormant LIC niche in the bone marrow endosteal region, i.e. TGFβ1 production and low perfusion. TGFβ1 and rapamycin maintained excellent cell viability. In contrast, serum withdrawal, the most common approach to dormancy induction in suspension culture, exacts a heavy toll on leukaemia cell viability that might overshadow pathways of dormancy maintenance [8]. Our choice of three day cell conditioning with TGFβ1 and rapamycin before GEP was informed by work documenting many differences between transient (induction of dormancy) and long-term (dormancy maintenance) gene expression profiles in somatic cells [25, 26]. Our work focuses on consequences of a 3-day exposure to dormancy inducers and gives time for an equilibrium to be established between oncogene-driven growth and niche-driven quiescence, allowing us to describe likely characteristics of the chemoresistant LIC in the niche. The up-regulation of many genes in dormant TF-1a cells despite the decrease in total RNA content indicates that dormancy is not merely an inhibition of proliferation or exit from the cell cycle, but is a phenomenon associated with active biological processes.

Upregulation of adhesion-related genes predominated in our model, and adhesion to bone marrow stromal cells, vitronectin and hyaluronic acid was also documented, supporting a role for recruitment and retention in specific microenvironments as a dominant characteristic of dormant LIC. Proliferating HSC and LSC display impairment of engraftment and reconstitution ability if compared to dormant counterparts [27, 28]. Enrichment for cell adhesion molecules has been documented in the HSC quiescence signature [29].

The most up-regulated gene in the dormancy signature was the osteopontin-coding gene SPP1. Osteopontin can function as adhesion protein, cytokine or chemokine and is implicated in HSC migration and self-renewal [30]. In a study of ALL cells, osteopontin caused the cells to exit the cell cycle and rendered them resistant to chemotherapy *in vivo*. The study did not identify a cell cycle effect for osteopontin *in vitro* and the authors concluded that osteopontin’s role was likely to be in regulating the homing of the leukaemia cells to the dormancy-inducing niche [31]. We demonstrated that osteopontin expression in AML blasts is dependent on tissue localisation, being high in biopsy material and low or negative in blasts from peripheral blood or bone marrow aspirates. *Intracellular* osteopontin can act as a bridging molecule for cytoskeletal re-arrangement and signal transduction [32, 33]. The cytoskeleton is likely affected by the increase in MYLK, which was the second most upregulated gene in our model. MYLK codes for a myosin light chain kinase that phosphorylates the microfilament protein NMM-II. MYH9, an isoform of NMM-II, is also upregulated in our model. This pathway is involved in sensing mechanical inputs from the extracellular environment and their transduction into intracellular signals and has many roles in the bone marrow niche [34]. MYLK overexpression has been reported in other models of dormancy initiation [26] and maintenance [25].

ITGB3 is an essential molecule for leukaemia cell propagation [17]. Its knockdown results in impairment of LSC homing (specifically to the bone marrow endosteal surface), downregulation of an LSC transcriptional programme, and induced differentiation [17]. ITGB3 is upregulated in the CD34^+^CD38^-^ blast subset [35] and has been implicated in chemoresistance [36]. Osteopontin can augment ITGB3-mediated spreading and signal transduction [20].

A critical feature of the dormant cell is resistance to differentiation [25]. In this context GEP showed upregulation of PLXN1, MMP2, GRP-56, TIM3, MEIS1 and its target RGS1, and ITGA6, all previously associated with a stem or multipotent progenitor stage of normal haematopoiesis [37], and the model also showed downregulation of differentiation associated genes CEBPA and CSF2RB. Several genes upregulated in the TF1a dormancy model have been described in leukaemia initiating cells, but appear not to be crucial for maintenance of normal HSCs, e.g. CD44 [18], ITGB3 [17], TIM3 (HAVCR2) [38] and IL3RA [39].

In addition to adhesion and stemness/inhibition of differentiation molecules we also documented tumour suppressor gene upregulation in TF-1a cells induced to dormancy (Figure 3). Strikingly, the only canonical cell cycle regulator upregulated in the model was the tumour suppressor CDKN2B (the cyclin-dependent kinase inhibitor p15). This gene is known to be epigenetically regulated, and is frequently mutated with a poor prognostic impact in AML [40]. In preliminary work we noted a transient induction of p57 by TGFβ1 (data not shown), which had disappeared by 24 hours. This reinforces the point made above that initiation and maintenance of dormancy can be characterised by largely different transcriptional programs. BTG2 upregulation may be a common dormancy feature. Classified as a tumour suppressor [41], it has also been documented to be overexpressed in somatic dormant and sublethally damaged cells [42].

This study focused on the profile of dormancy induced by extrinsic factors. In the case of SPP1/osteopontin, we documented strong expression only in bone marrow biopsies or mononuclear cell isolates that had been cultured with TGFβ1. Nevertheless it was interesting to establish that TCGA primary AML samples have varied expression of dormancy signature genes and that these distributed into clusters unrelated to cytogenetic risk group. ITGB3 (discussed above) was upregulated in the dormancy model and also associated with poor one year survival in TCGA samples.

In this study, we demonstrated an *in vitro* approach mimicking key attributes of the LIC niche to characterize dormancy in CD34^+^CD38^-^ AML cells. Genes identified in this model are candidates for investigations to establish potential targets for rooting out dormant AML cells.

## MATERIALS AND METHODS

### AML cell lines and cell culture

TF-1a cells were from The European Collection of Animal Cell Culture (Salisbury, UK) and were cultured in RPMI-1640 with 10% FCS and glutamine. Following treatments, cells were counted flow cytometrically using an internal standard as described [43]. Testing to authenticate the cell line was performed at the last passage of each thawed batch using multiplex short tandem repeat analysis (Powerplex 16, Promega, Southampton, UK). Mycoplasma testing was carried out routinely using the Mycoalert mycoplasma detection kit (Lonza, Rockland, USA) and following the manufacturer’s instructions.

### Patient cells

The investigation was conducted on samples obtained with informed consent in accordance with ethical approval (Nottingham Research Ethics Committee 1) and Declaration of Helsinki and according to international guidelines and has been approved by the authors' institutional review board.

### Indirect immunofluorescence assay for FOXO3a

Unmanipulated and TGFβ1-treated TF-1a cells were allowed to adhere on Poly-D-Lysine (PDL, p6407 Sigma)-coated glass coverslips. They were stained with the 75D8 anti-FOXO3A antibody from Cell Signalling Technologies. Images were acquired using an Olympus fluorescence microscope and visualised with ImageJ software.

### Immunohistochemistry analysis of human bone marrow biopsies

The expression of osteopontin (OPN) was investigated by immunohistochemistry (IHC) analysis of formalin-fixed paraffin-embedded BM biopsy sections using standard procedures with mouse anti-human monoclonal anti-OPN (ab166709; abcam) and the X-Cell Plus Universal Polymer horseradish peroxidase (MP-XCP-U25; Menarini Diagnostics) detection system.

### Flow cytometry-based assays

Flow cytometry analyses were carried out using the FACS Canto II (BD Biosciences) with Diva software. Surface marker immunophenotyping was carried out using fluorochrome-conjugated anti-human monoclonal antibodies or isotype controls (BD Biosciences). The ALDEFLUOR™ reagent kit (Stem Cell Technology #01700) was used to measure the intracellular activity of aldehyde dehydrogenase (ALDH). Cell viability was measured using Annexin V labelling (Trevigen Annexin V kit, #4830-250-K). Mouse anti-human anti-Ki-67-FITC (#556026; BD Pharmingen, Oxford, UK) was used for detecting the nuclear protein Ki-67 in fixed and permeabilized AML cells. The protocol was based on a previously published method [44].

### Adhesion to cells or to immobilised molecular substrates assays

HS-5 stromal cells [45] (American Type Culture Collection) were grown in DMEM with 10% FCS. Untreated and treated TF-1a cells were co-incubated with confluent HS-5 for three hours and detached by trypsinisation. TF-1a cells were identified and counted as described (Supplementary Figure 7).

96 well plates were coated with 10 μg/ml fibronectin, 10 μg/ml vitronectin or 1 mg/ml hyaluronic acid for 2 hours at 37^°^C, rinsed twice in PBS and dried overnight. TF-1a cells were allowed to attach for three hours and non-adherent cells were removed by washing with warm PBS. Attached cells were counted with a light microscope fitted with a wire grid (0.9X0.9 mm squares, quadruplicate plates).

### Real-time quantitative polymerase chain reaction (qPCR) assay

Quantitative PCR was performed as previously described [46] using primer sequences as listed in Supplementary Table 10 and standardised with β2-microglobulin (B2M) [47].

### Global human genome gene expression profile (GEP) assay

RNA was processed according to the manufacturer’s protocol using the Agilent 2100 Bioanalyzer and Human Gene 2.1 Array Strips (#902114; Affymetrix) with the microarray system GeneAtlas from Affymetrix.

### Data analysis

GEP data was analysed using Partek Genomics Suite 6.6 software. For the analysis of patient outcomes and groupings, the TCGA LAML data set GSE68833 was downloaded from the Gene Expression Omnibus. Hierarchical clustering, using the Ward’s method was done with the Partek software. Additional analyses were performed using the Statistical Package for Social Sciences, version 23 (SPSS, Chicago, IL, USA).

## SUPPLEMENTARY MATERIALS FIGURES AND TABLES











## Abbreviations

ALDH: aldehyde dehydrogenase
ALL: acute lymphoblastic leukaemia
AML: acute myeloid leukaemia
Β2M: beta 2 microglobulin
BM: bone marrow
BTG2: B cell translocation gene 2
CDKN2B: cyclin-dependent kinase inhibitor 2B
CEBPA: CCAT enhancer binding protein alpha
CSF2RB: colony stimulating factor 2 receptor beta
DMEM: Dulbecco Modified Eagle Medium
FAB: French American British
FACS: fluorescence activated cell scanning
FCS: foetal calf serum
FDR: false discovery rate
FITC: fluorescein isothiocyanate
FLT3: fms-related tyrosine kinase 3
FOX03A: forkhead box 03A
GEP: gene expression profiling
GO: gene ontology
GRP-56: G protein-coupled receptor 56
HSC: haematopoietic stem cells
IL3RA: interleukin 3 receptor alpha
IHC: immunohistochemistry
ITGB3: integrin beta 3
ITGA6: integrin alpha 6
ITGAV: integrin alpha v
IL6ST: interleukin 6 signal transducer
KEGG: Kyoto Encyclopaedia of Genes and Genomes
LIC: leukaemia-initiating cells
MEIS1: myeloid ecotropic viral integration site 1
MMP2: matrix metalloproteinase 2
MTOR: mammalian target of rapamycin
MYH9: myosin heavy chain 9
MYLK: myosin light chain kinase
NPM1: nucleophosmin 1
OPN: osteopontin
PBS: phosphate buffered saline
PCR: polymerase chain reaction
PLXN1: plexin A1
PTH2: parathyroid hormone 2
RGS1: regulator of G-protein signalling 1
RNA: ribonucleic acid
RPMI: Roswell Park Memorial Institute
SPP1: secreted phosphoprotein 1
TCGA: the cancer genome atlas
TGFβ1: transforming growth factor beta 1
TIM3: T cell immunoglobulin and mucin domain 1
