# Supplementary material for: A molecular signature of dormancy in CD34+CD38- acute myeloid leukaemia cells
Source: Oncotarget. 2017 Nov 30;8(67):111405–18. doi: 10.18632/oncotarget.22808 (PMC5762331; doi:10.18632/oncotarget.22808)
Supplement: Supplementary file 1 [file oncotarget-08-111405-s001.pdf]

A molecular signature of dormancy in CD34$^+$CD38$^-$ acute myeloid leukaemia cells

SUPPLEMENTARY MATERIALS

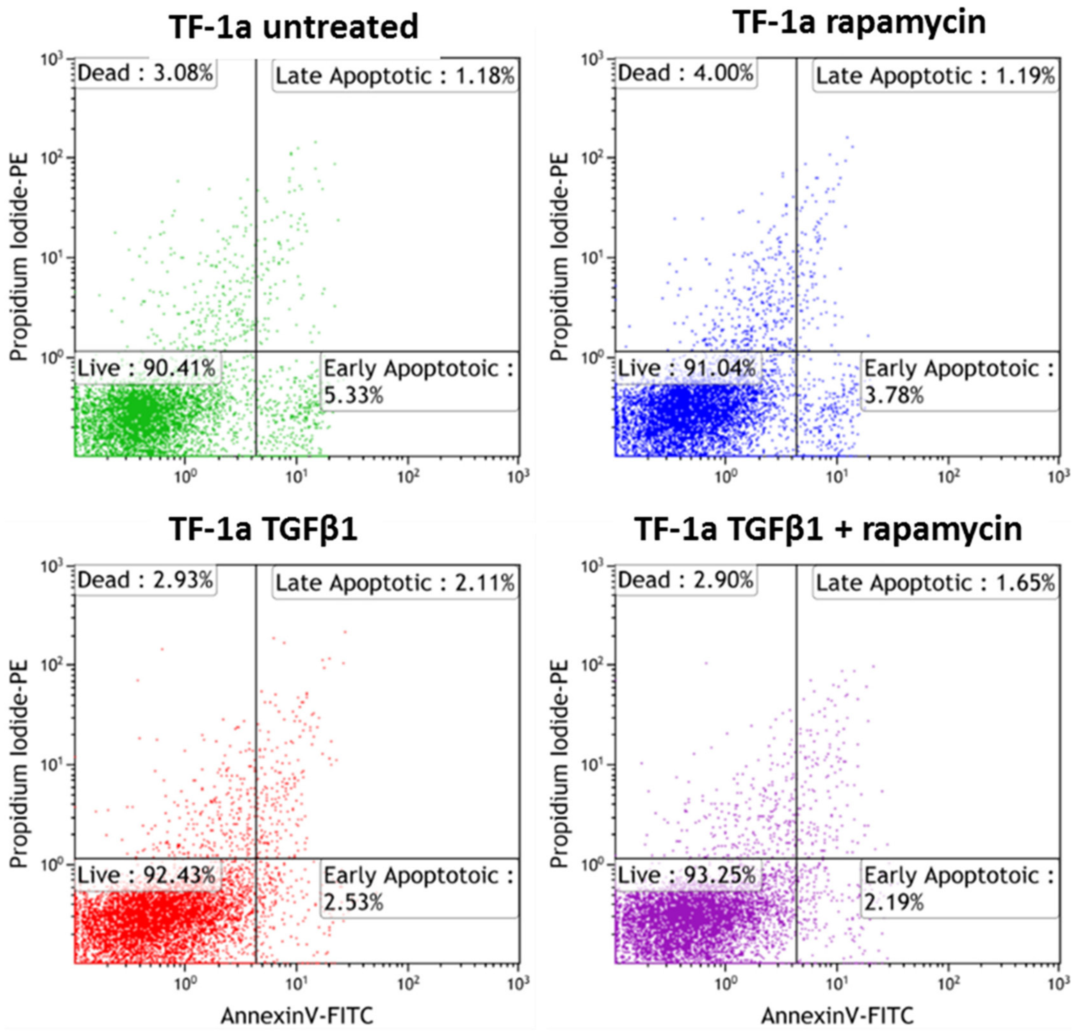

Supplementary Figure 1: TF-1a cell viability. Cells were probed with AnnexinV and Propidium Iodide at the end of the incubation period of 3 days culture with 4ng/ml of TGFβ1 or 100nM of Rapamycin or both and subjected to flow cytometric analysis. The plots are representative examples of three independent experiments that showed that the conditioning was not causing apoptosis.

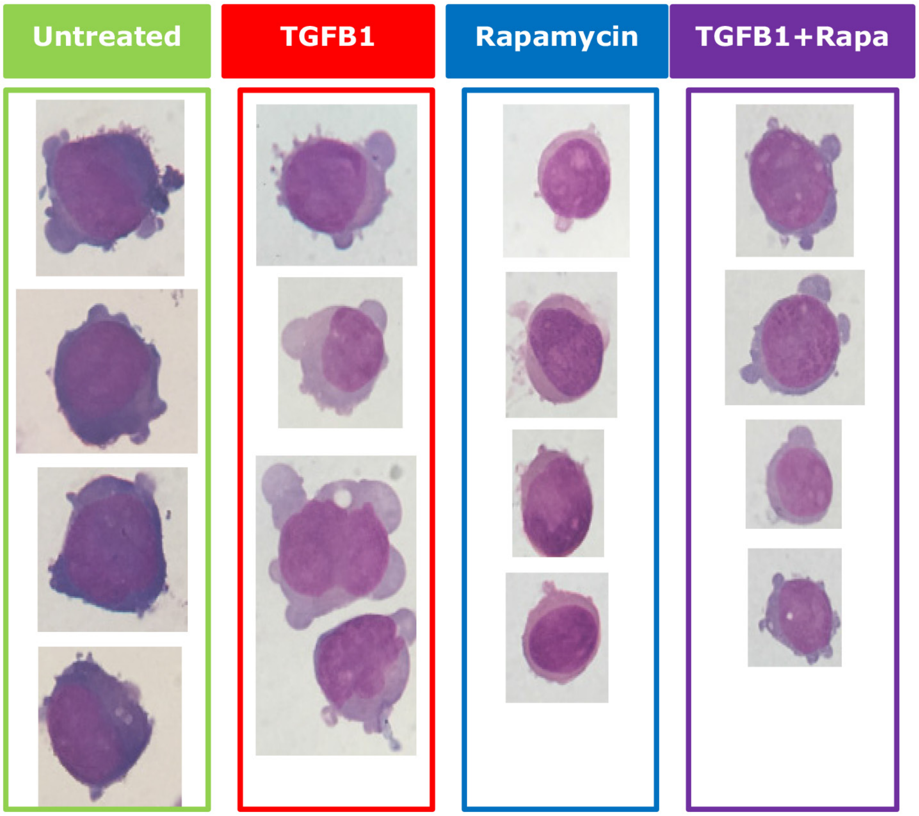

Supplementary Figure 2: TF-1a Cell line Morphology. The TF-1a Cell line Morphology was assessed before (Cycling TF-1a) and after (Dormant TF-1a) 3 days culture with 4ng/ml of TGFβ1 or 100nM of rapamycin or both. Air-dried smears of TF-1a cells of each condition were stained with Wright-Geimsa stain and examined and pictured using Olympus fluorescence microscope adjusted as a light microscope. Images were processed with Image J software. The morphology indicates that an undifferentiated phenotype is preserved with the conditioning treatments.

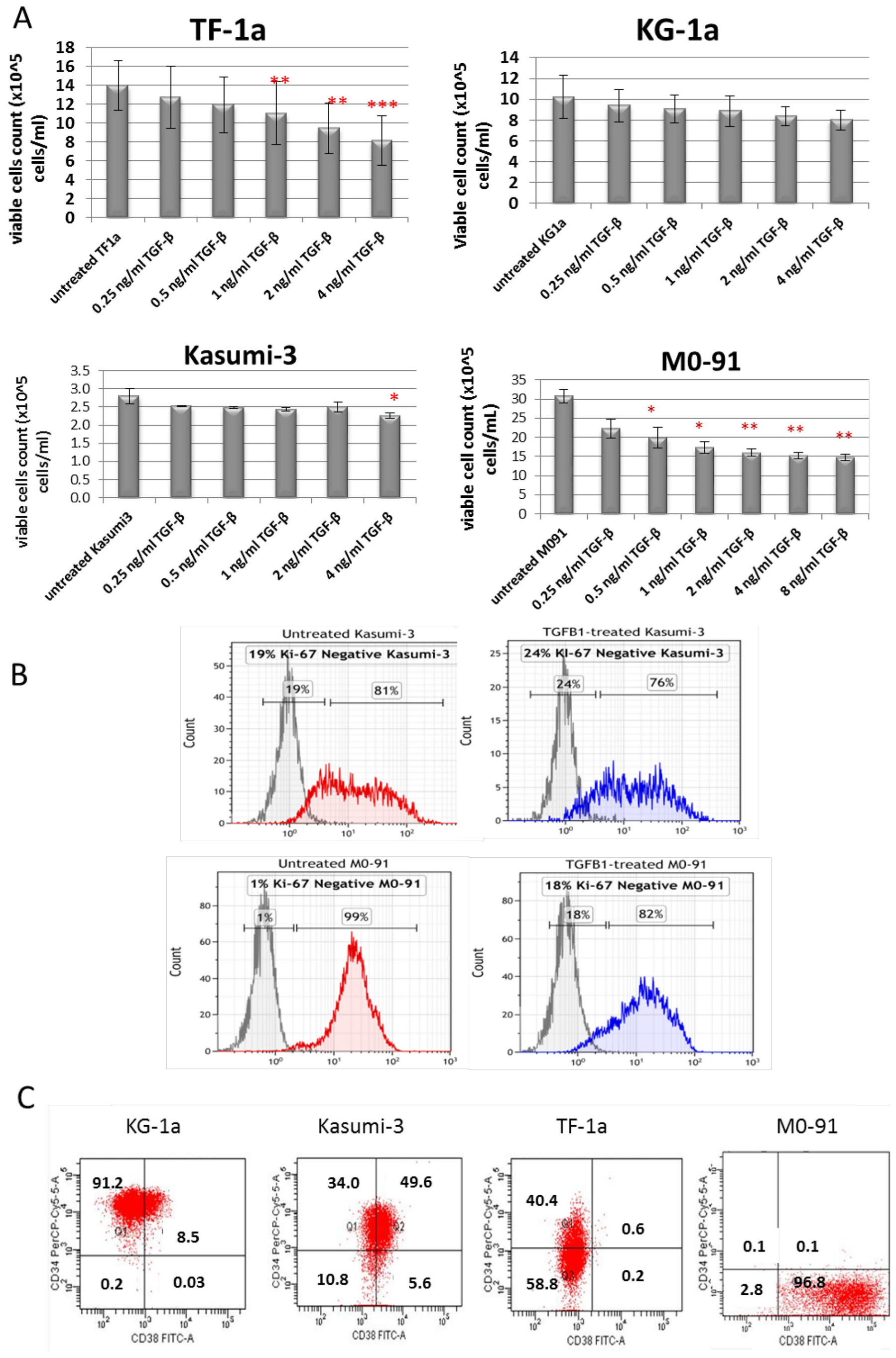

Supplementary Figure 3: The inhibitory effect of TGFβ1 on TF-1a cell growth and comparisons with additional cell lines. (A). Effect of escalating doses of TGFβ1 on the proliferation of four cell lines. All cells were seeded at a concentration of 2x10^5 cells/ml and incubated in suspension culture for 72 hrs with TGFβ1. The columns represent mean values of three independent experiments. The error bars represent +/- 1SD. (*: $p < 0.05$; **: $p < 0.01$; ***: $p < 0.001$). **(B)** After eliminating the non-significantly-responding KG-1a from the analysis, Ki-67 was measured in the three remaining cells lines after 72 hrs with TGFβ1. Results are illustrated for the M0-91 and Kasumi-3 cell lines. (TF-1a results are shown in Figure 1C.) The figure illustrates an example of at least 3 biological replicates showing similar results. **(C)** CD34 and CD38 status of KG1-a, Kasumi-3, TF-1a and M0-91 AML cell lines. Values shown are the percentage of positive cells in each quadrant of the FACS plots.

Note: KG-1a were purchased from The European Collection of Animal Cell Culture (Salisbury, UK) while Kasumi-3 was purchased from The American Collection of Animal Cell Culture (USA) and M0-91 was a gift from Dr. Joseph M. Scandura (Sloan-Kettering Institute, USA) and were cultured as described for TF-1a, except that 20% FCS was used with KG-1a and Kasumi-3 cells.

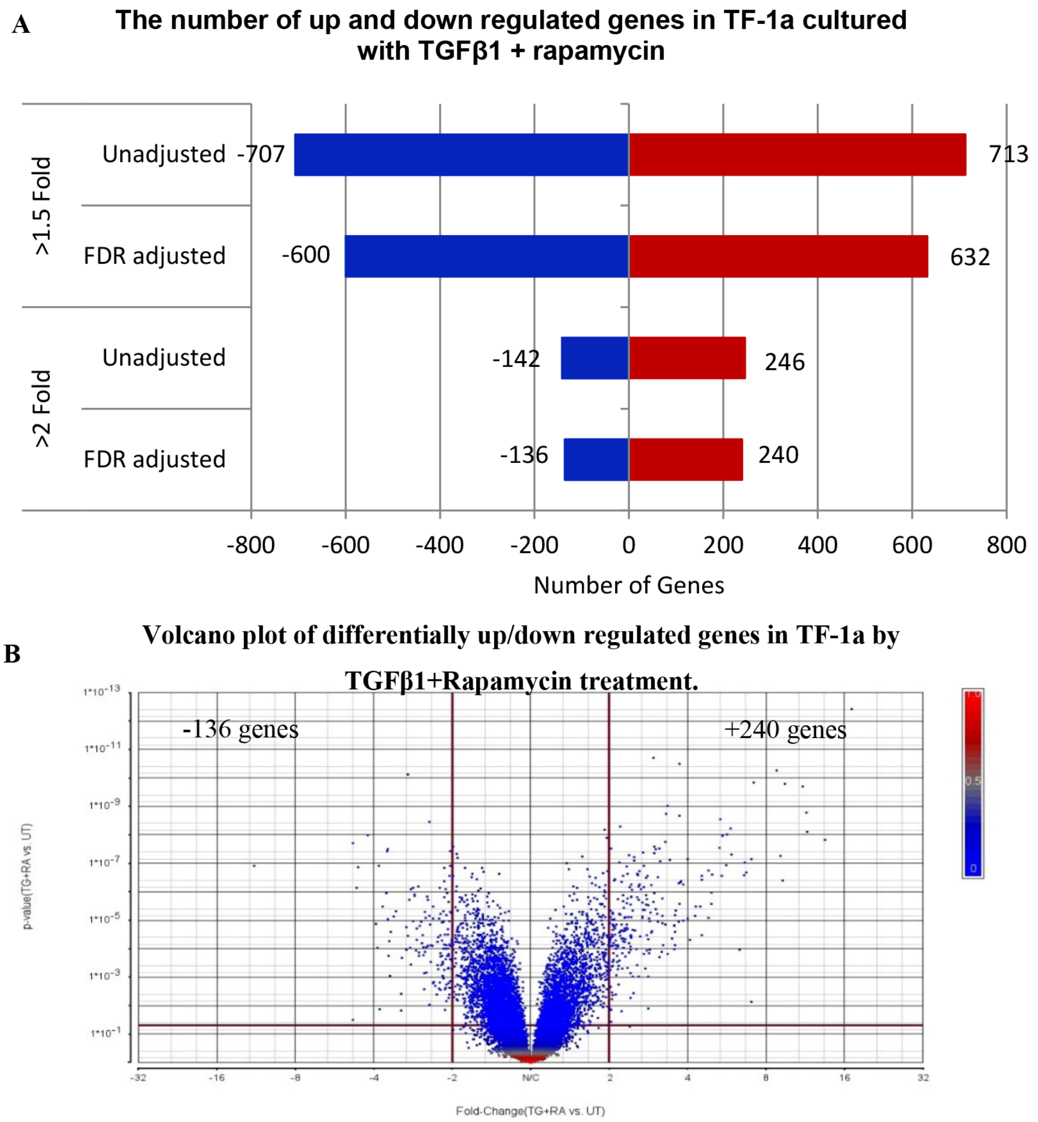

Supplementary Figure 4: Overview of gene expression profiling results. (A) The bars represent the number of genes that were differentially up- (dark red) or down- (blue) regulated in TF-1a cells cultured for 3 days with 4ng/ml of TGFβ1 and 100nM of rapamycin in comparison to their untreated counterparts, distributed according to fold change and un-adjusted or false discovery rate (FDR)- adjusted p-value (<0.05). **(B)** The volcano scatter-plot illustrates all differentially up/down regulated genes in dormant TF-1a cells distributed according to the statistical significance (Y axis) and fold change (X axis). Each dot represents a gene. The vertical lines represent the 2 fold change cut-off limit and the horizontal line represent the selected p value to determine the list of genes of interest.

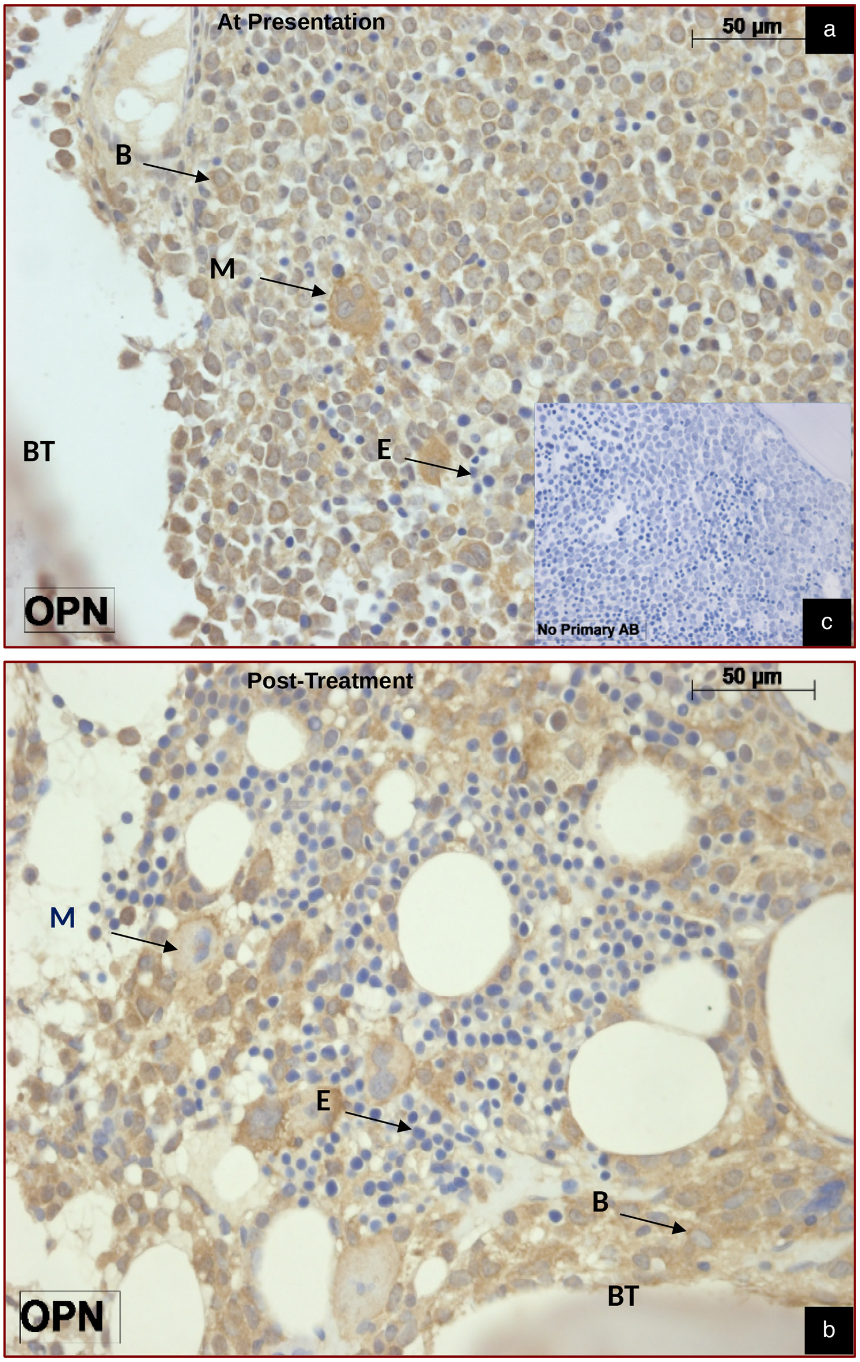

Supplementary Figure 5: Osteopontin expression in BM biopsies of AML patients. The pictures compare osteopontin expression by IHC in presentation (picture **a**) and post-treatment (picture **b**) BM biopsies for the same patient (patient number 5 in Supplementary Table 6). Notably, despite the signs of marrow regeneration post treatment, which is represented by the active erythropoiesis and megakaryopoiesis, residual leukemic blasts retaining osteopontin expression can be seen near the bone trabeculae. No primary antibody negative control (pictures **c**) were used to validate the IHC staining. B, blast; E, erythrocyte; M, megakaryocyte; BT, bone trabeculae.

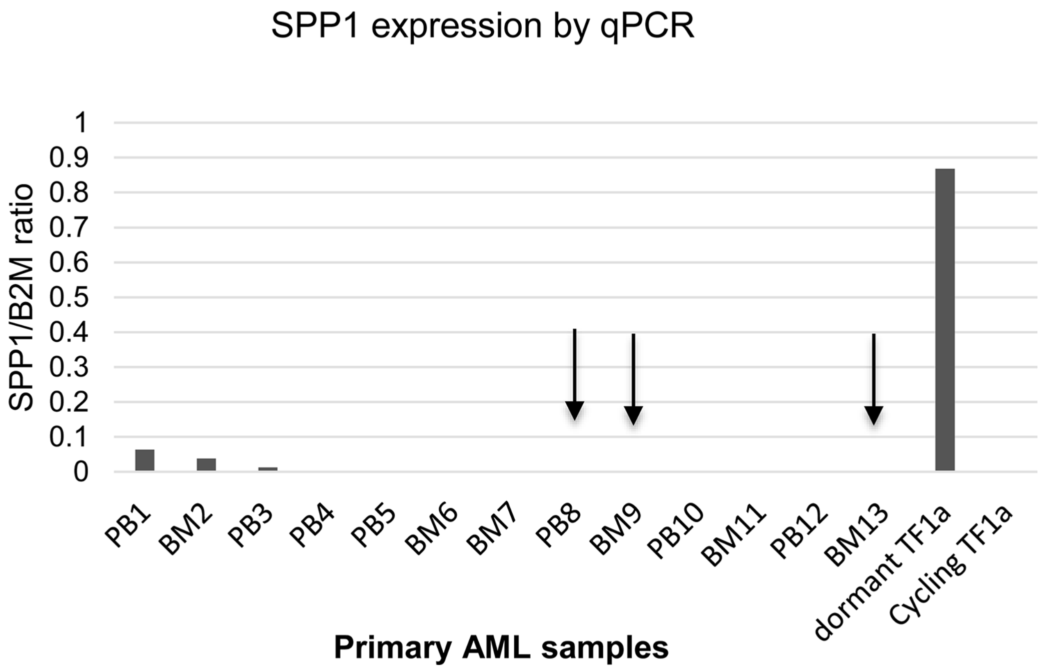

Supplementary Figure 6: SPP1 expression in leukaemic blasts. SPP1 message was measured by qPCR in 13 CD2-depleted pre-treatment cases of AML, comprising 7 peripheral blood (PB) and 6 bone marrow aspirate (BM) samples. Mononuclear cell samples from 3 patients whose biopsy trephines had been analysed were available in our bank, and these are marked with arrows on the graph (none of the three had detectable expression in the isolated mononuclear cells). TF-1a were re-processed concurrently as positive and negative controls.

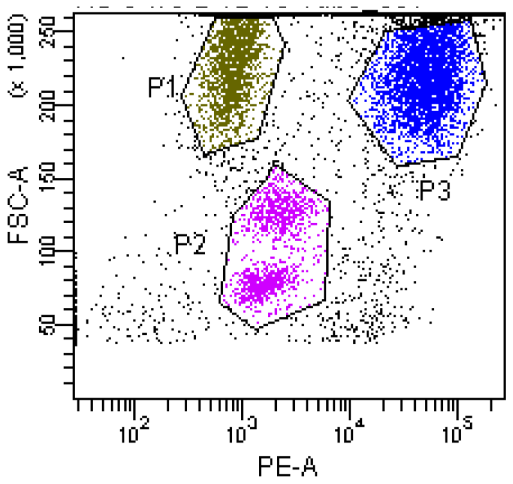

Supplementary Figure 7: TF1-a adherence to HS-5 stromal cells. A schematic diagram for quantitative measurement of TF1-a adherence to HS-5 stromal cells. An adhesion assay was performed as described in the methods. Harvested adherent TF1-a cells (CD73-PE negative, forward scatter high, P1 in flow cytometry dot plot) were counted. P3 identifies CD73+ stromal cells. P2 identifies the internal standard for counting (reference 43).

Supplementary Table 1: The 240 significantly upregulated genes in dormant TF1-a cells

See Supplementary File 1

Supplementary Table 2: The 136 significantly downregulated genes in dormant TF1-a cells

See Supplementary File 2

Supplementary Table 3: Canonical TGFβ1 and mTORC1 pathways are significantly affected in the dormancy model

Process	GO term	upregulated genes			downregulated genes		
		gene symbol	FDR q value	enrichment score	gene symbol	FDR q value	enrichment score
**TGFbeta signalling**	GO: 0017015		1.21E-03	7.25	no significant change		
		FOLR1					
		SKIL					
		TGFB1					
		SMAD6					
		SMAD7					
		BAMBI					
		ITGA3					
		CDKN2B					
**cholesterol metabolic process***	GO: 0008203	no significant change				5.04E-03	12.07
					LSS		
					INSIG1		
					HMGCS1		
					STAR		
					PCSK9		
					MVK		
					CEBPA		
					APOC1		

Cells were treated with 4 ng/ml TGFβ1 and 100 nM rapamycin for 3 days and then analysed by gene expression profiling. Enrichment analysis was carried out using the GOrilla platform (cbl-Gorilla.cbs.technion.ac.il).
*Cholesterol metabolism has been used as a major transcriptional target pathway of mTORC1 (Duvel 2010).
Reference: Duvel K, Yecies JL, Menon S, Raman P, Lipovsky AI, Souza AL, Triantafellow E, Ma Q, Gorski R, Cleaver S, Vander Heiden MG, MacKeigan JP, Finan PM, Clish CB, Murphy LO and Manning BD. Activation of a metabolic gene regulatory network downstream of mTOR complex 1. Molecular cell. 2010; 39(2):171-183. doi:10.1016/j.molcel.2010.06.022.

Supplementary Table 4: The significantly enriched biological processes/signalling pathways in the 240 genes upregulated genes in dormant AML cells*

See Supplementary File 3

Supplementary Table 5: The significantly enriched biological processes/signalling pathways in the 136 downregulated genes in dormant AML cells*

See Supplementary File 4

Supplementary Table 6: The clinical characteristics of the 14 BM biopsies of the 7 AML patients investigated in this study

Patient Code	age	gender	immunophenotyping	cytogenetics	molecular genetics	type and duration of induction treatment	disease status at time of biopsy
**1P**	67	F	31% CD34, CD117pos, aberrant CD7, cytCD79a	46XXdel(2)(q11q21),der(7) t(1;7)(q2q22), add(11)(q2)[9]v	FLT3 ITD	presentation	presentation
**1T**	67	F	No data	Abnormal as previous, but donor cells also present	No data	post-transplant	incomplete engraftment and disease progression
**2P**	45	M	CD34neg/ CD117neg54% CD64+ monocytes consistent with CMML in transformation	NORMAL	NORMAL	presentation	Presentation. AML, consistent with transformed MDS or CMML. Longstanding monocytosis
**2T**	45	M	No data	No data	No data	post one cycle chemo	probable residual disease
**3P**	78	M	36% CD34+, CD117+, aberrant CD7	NORMAL	all neg.	presentation	presentation
**3T**	78	M	69% CD34+, CD117+, aberrant CD7	No data	No data	post 4 cycles low dose ara C	resistant disease
**4P**	83	F	CD34neg, CD117pos, aberrant CD7	NORMAL	FLT3 ITD	presentation	presentation
**4T**	83	F	No data	No data	No data	post 2 cycles low dose araC	partial response
**5P**	9	M	No data	NORMAL	No data	No data	relapse
**5T**	9	M	No data	No data	No data	chemo (no details)	remission
**6P**	25	F	44% CD34+	minus 7 (9/10)	all neg.	presentation	presentation, biopsy suggests AML on an MDS background
**6T**	25	F	No data	Normal, no sign of chr 7 abn. by FISH	No data	post FLAG-Ida	normal regenerating marrow
**7P**	60	F	20% CD34+, aberrant cytCD79a	NORMAL	FLT3ITD	presentation	presentation
**7T**	60	F	20% CD34+	No data	No data	post chemo), falling counts	Relapse

Supplementary Table 7: Functional group enrichment analysis of TCGA gene clusters mapped by unsupervised hierarchical clustering

	number of genes in cluster	Adhesion related number of genes altered (enrichment score)	Stemness and differentiation related number of genes altered (enrichment score)	tumour suppression related number of genes altered (enrichment score)
**CLUSTER1**	65	2 (0.43)	4 (0.96)	5 (1.11)
**CLUSTER 2**	84	8 (1.33)	5 (0.93)	6 (1.03)
**CLUSTER 3**	108	8 (1.03)	12 (1.74)*	10 (1.34)
**CLUSTER 4**	97	8 (1.15)	9 (1.45)	13 (1.94)*
**CLUSTER 5**	128	15 (1.63)*	8 (0.98)	12 (1.36) *
**Functional group total**		**27**	**24**	**26**

The genes mapped to the five groups of genes illustrated in Figure 7A were intersected with the three functional groups of genes listed in Figure 3. The number of altered genes in each cluster (b) was divided by the total number of genes in the cluster (n). The total number of genes in each functional group B was divided by 376 (N, the total number of differentially regulated genes in the dormancy model). An enrichment ratio (b/n)/(B/N) was then calculated.

* indicates statistically significant enrichment (Fisher's exact test).

Supplementary Table 8: Clinical and laboratory characteristics of the 149 TCGA patients in dormancy cluster 4 compared to clusters 1-3

		All	Clusters 1-3	Cluster 4	Cluster Comparison
**Number**		149	106 (71)	43 (29)	
					**Mann Whitney P value**
**Age**	median		58	57	
	interquartile range		44-65	45-67	NS
**WBC**	median		12.1	16.9	
	interquartile range		2.8-47.2	4.3-48.5	NS
		**All - number**	**Clusters 1-3 number (percent)**	**Cluster 4 number (percent)**	**χ^2 P value**
**Gender**	male	81	51 (48)	30 (70)	
	female	68	55 (52)	13 (30)	0.019
**Cytogenetic group**	favourable	22	18(17)	4(10)	
	intermediate	92	62(59)	30(73)	
	adverse	32	25(24)	7(17)	
	total	146	105	41	NS
**FAB**	M0	11	9(9)	2(5)	
	M1	38	31(31)	7(16)	
	M2	35	23(23)	12(28)	
	M4	35	24(24)	11(26)	
	M5	19	14(14)	5(12)	
	M6	3	0(0)	3(7)	
	M7	3	0(0)	3(7)	
	total	144	101	43	0.005
**Induction**	intensive	127	89 (84)	38 (90)	
	non-intensive	20	17 (16)	3 (7)	
	total	147	106	41	0.032
**FLT3**	wildtype	103	71 (68)	32 (84)	
	mutant	40	34 (32)	6 (16)	
	Total	143	105	38	.051
**NPM1**	wildtype	108	75 (71)	33 (79)	
	mutant	39	30 (29)	9 (21)	
	total	147	105	42	NS

Supplementary Table 9: Variables entered into multivariate logistic regression for 1 year survival in 106 intensively treated TCGA patients

		All	*Alive*	*Dead*	*P value on final step*
			Number (%)	*Number (%)*	*logistic regression*
		106	*61 (58)*	*45 (42)*	
**CYTOGENETIC GROUP**					
	favourable	13	10 (16)	3 (7)	
	intermediate	64	37 (61)	27 (63)	
	adverse	27	14 (23)	13 (30)	
	total	*104*	*61*	*43*	*0.412*
**FLT3**	*wildtype*	76	46 (60)	30 (39)	
	mutant	29	14 (48)	15 (31)	
	total	*105*	*60*	*45*	*0.219*
**NPM1**	*wildtype*	75	43 (72)	32 (71)	
	mutant	30	17 (28)	13 (29)	
	total	*105*	*60*	*45*	*0.709*
**GENES OVER-EXPRESSED IN CLUSTER 4**					
	PTH2	106	61 (58)	45 (42)	0.028
	ITGB3	106	61 (58)	45 (42)	0.021
	IL3RA	106	61 (58)	45 (42)	0.486
	STX1A	106	61 (58)	45 (42)	0.903
	GAL	106	61 (58)	45 (42)	0.897
	KLRC3	106	61 (58)	45 (42)	0.582
	IL6ST	106	61 (58)	45 (42)	0.022
	LTBP3	106	61 (58)	45 (42)	0.869
	GPR153	106	61 (58)	45 (42)	0.477
	ANGPT2	106	61 (58)	45 (42)	0.641
**AGE**		106	61 (58)	45 (42)	
			(median age 55)	(median age 57)	0.974

Supplementary Table 10: List of qPCR primers

Target Gene (Transcript)		Primer Sequence	Product size in bp	Exon/Exon Junction(primer)	Supplier
β2-microglobulin	F	5'-GAGTATGCCTGCCGTGTG-3'	110	N/A	Invitrogen
	R	5'-AATCCAAATGCGGCATCT-3'			
SPP1(NM_001251830)	F	5'-AGTTCTGAGGAAAAGCAGCACT-3'	102	258/259(F)	Invitrogen
	R	5'-GTCCAAGCTTCTGGGGACAA-3'			
MYLK(NM_053025)	F	5'-CTGGCTCCCATTACCTCTGC-3'	127	1798/1799(R)	Invitrogen
	R	5'-ACGGCAAGCCTTTCCACTTG-3'			
PLXNC1(NM_005761)	F	5'-GAGATACGTGACGGCTTTGCT-3'	134	3232/3233(F)	Invitrogen
	R	5'-TGGGTGAAGCCACCTGACTC-3'			
ITGB3(NM_000212)	F	5'-AATGGGACACAGCCAACAAC-3'	121	2321/2322(F)	Invitrogen
	R	5'-ATCGTGGCACAGGCTGATAA-3'			
ITGB4(NM_001005619)	F	5'-TCTCTGGGACCTTGGCAAAC-3'	89	87/88(F)	Invitrogen
	R	5'-GCAGTAGGCGCAGTCCTTAT-3'			
ITGA3(NM_002204)	F	5'-TGCCTACAACTGGAAAGGAAAC-3'	126	1136/1137(F)	Invitrogen
	R	5'-CTGCCTACCTGCATCGTGTA-3'			
CD44(NM_000610)	F	5'-CAAGCACAATCCAGGCAACT-3'	161	1587/1588(F)	Invitrogen
	R	5'-ATGGCTGGTATGAGCTGAGG-3'			
