# Supplementary material for: A molecular signature of dormancy in CD34+CD38- acute myeloid leukaemia cells
Source: Oncotarget. 2017 Nov 30;8(67):111405–18. doi: 10.18632/oncotarget.22808 (PMC5762331; doi:10.18632/oncotarget.22808)
Supplement: Supplementary file 2 [file oncotarget-08-111405-s002.docx]

**Supplementary table S1:** The 240 significantly upregulated genes in dormant TF1-a cells

Genes marked with an asterisk are grouped in Figure 3:-

Adhesion related *

Stemness and differentiation-related *

Tumour-suppressor and proliferation regulator genes *

|  | **Gene Symbol** | **Fold-Change** |  | **Gene Symbol** | **Fold-Change** |  | **Gene Symbol** | **Fold-Change** |
| --- | --- | --- | --- | --- | --- | --- | --- | --- |
| **1** | SPP1 * | 17.1 | **81** | ANGPTL2 | 2.9 | **161** | LOC100133920 | 2.3 |
| **2** | TMC7 | 13.5 | **82** | PRSS1 | 2.9 | **162** | KLHL41 | 2.3 |
| **3** | ABCC3 * | 11.5 | **83** | VDR** | 2.9 | **163** | GRAMD2 | 2.3 |
| **4** | PTPRU** | 11.4 | **84** | MYOM1 | 2.9 | **164** | CLU | 2.3 |
| **5** | MYLK * | 11 | **85** | SP7 | 2.8 | **165** | SCARA3 | 2.3 |
| **6** | FLJ35282(DMRTA1) | 9.5 | **86** | SLC22A23 | 2.8 | **166** | RGS10 | 2.3 |
| **7** | HTN3* | 9.3 | **87** | QPCT | 2.8 | **167** | IL16 | 2.3 |
| **8** | ALOX5AP | 9.1 | **88** | SYNPO2 | 2.8 | **168** | CD34** | 2.3 |
| **9** | PLXNC1*** | 8.8 | **89** | GAL* | 2.8 | **169** | YPEL3 | 2.3 |
| **10** | ITGB3 ** | 7.2 | **90** | GRAP2 | 2.8 | **170** | ALOX12* | 2.3 |
| **11** | ADAMTS10 | 7.1 | **91** | CEP112 | 2.8 | **171** | KLHL24 | 2.2 |
| **12** | RGS9 | 6.7 | **92** | ITGAV* | 2.8 | **172** | GOLGA6L5 | 2.2 |
| **13** | PTH2 | 6.6 | **93** | EBI3 | 2.8 | **173** | KIAA1161 | 2.2 |
| **14** | SMAD7* | 6.6 | **94** | CMTM1 | 2.8 | **174** | C7orf41 | 2.2 |
| **15** | CCL2 * | 6.3 | **95** | ITGA6* | 2.8 | **175** | SERPINF1 | 2.2 |
| **16** | IL3RA* | 5.9 | **96** | RSPH1 | 2.8 | **176** | SLC35D2 | 2.2 |
| **17** | SYTL5 | 5.9 | **97** | PROCR* | 2.8 | **177** | LOC642361 | 2.2 |
| **18** | IL3RA* | 5.7 | **98** | CRLF2 | 2.7 | **178** | BMF | 2.2 |
| **19** | CNN1* | 5.6 | **99** | KIAA1109 | 2.7 | **179** | ACSM3 | 2.2 |
| **20** | CNR1* | 5.5 | **100** | GRK5 | 2.7 | **180** | MEF2A | 2.2 |
| **21** | MMP2* | 5.4 | **101** | C19orf77 | 2.7 | **181** | EEPD1 | 2.2 |
| **22** | CNR1 | 5.4 | **102** | ABAT | 2.7 | **182** | ARHGAP6 | 2.2 |
| **23** | MYO7A | 5.3 | **103** | BHLHE40 | 2.7 | **183** | KIAA1107 | 2.2 |
| **24** | CHST15 | 5 | **104** | BMPR2 | 2.7 | **184** | TPM1* | 2.2 |
| **25** | ITGB4 * | 4.9 | **105** | PLD4 | 2.7 | **185** | RASA4 | 2.2 |
| **26** | RASGRP1 | 4.9 | **106** | CDKN2B* | 2.7 | **186** | CD207 | 2.2 |
| **27** | NTRK3 | 4.8 | **107** | TCP11L2 | 2.7 | **187** | TSPAN9 | 2.2 |
| **28** | CPNE2 | 4.8 | **108** | GYPB | 2.6 | **188** | CNR1 | 2.2 |
| **29** | CYTIP | 4.5 | **109** | RD3L | 2.6 | **189** | INPPL1** | 2.2 |
| **30** | SLC4A1 | 4.5 | **110** | GPRC5B | 2.6 | **190** | EPHA4 | 2.2 |
| **31** | UNC5B | 4.5 | **111** | MEIS1* | 2.6 | **191** | ITGB5* | 2.2 |
| **32** | ITGA3 ** | 4.5 | **112** | ASAP2 | 2.6 | **192** | RD3 | 2.2 |
| **33** | SLC2A12 | 4.3 | **113** | TM6SF1 | 2.6 | **193** | HBE1 | 2.1 |
| **34** | LURAP1L | 4.3 | **114** | PLEKHH2 | 2.6 | **194** | PNMT | 2.1 |
| **35** | HAVCR2 (TIM-3) * | 4.1 | **115** | MMRN1* | 2.6 | **195** | LOC401321 | 2.1 |
| **36** | IDS | 4 | **116** | RASA4B | 2.6 | **196** | ENPP6 | 2.1 |
| **37** | SMAD6 | 4 | **117** | CCBP2 | 2.6 | **197** | S100A13 | 2.1 |
| **38** | NKD1 | 3.9 | **118** | FAM214A | 2.6 | **198** | ADC | 2.1 |
| **39** | VASH1* | 3.8 | **119** | CORO6 | 2.5 | **199** | SFXN3 | 2.1 |
| **40** | SPRY3 | 3.8 | **120** | GPR37 | 2.5 | **200** | USP18 | 2.1 |
| **41** | ENKUR | 3.8 | **121** | GFOD1 | 2.5 | **201** | BAMBI | 2.1 |
| **42** | SKIL* | 3.7 | **122** | GLIPR1 | 2.5 | **202** | TRPC3 | 2.1 |
| **43** | CD44** | 3.7 | **123** | PPBP | 2.5 | **203** | KIAA0355 | 2.1 |
| **44** | ECM1 | 3.7 | **124** | C15orf65 | 2.5 | **204** | TGFB1* | 2.1 |
| **45** | SPRY3 | 3.7 | **125** | IGFBP5 | 2.5 | **205** | Sep-04 | 2.1 |
| **46** | GSN | 3.7 | **126** | TNNI3 | 2.5 | **206** | HIVEP2 | 2.1 |
| **47** | SERPINE1 | 3.6 | **127** | KCNH3 | 2.4 | **207** | RAB30 | 2.1 |
| **48** | CLCA1 | 3.6 | **128** | MICAL3 | 2.4 | **208** | SEPT5-GP1BB | 2.1 |
| **49** | EPHB6 | 3.6 | **129** | GPR153 | 2.4 | **209** | TMEM158* | 2.1 |
| **50** | SIGLEC6* | 3.6 | **130** | GPR56** | 2.4 | **210** | MYEOV | 2.1 |
| **51** | FBXO32* | 3.6 | **131** | HRC | 2.4 | **211** | CMTM3 | 2.1 |
| **52** | DMTN | 3.6 | **132** | MRAP2 | 2.4 | **212** | THBS3* | 2.1 |
| **53** | DUSP21 | 3.5 | **133** | BTBD19 | 2.4 | **213** | EFHB | 2.1 |
| **54** | PTHLH* | 3.5 | **134** | CTSE | 2.4 | **214** | OPTN | 2.1 |
| **55** | SLC37A1 | 3.4 | **135** | BTG2* | 2.4 | **215** | KLRC3 | 2.1 |
| **56** | FMNL3 | 3.3 | **136** | SLC46A3 | 2.4 | **216** | IL10RB-AS1 | 2.1 |
| **57** | PLCL1 | 3.3 | **137** | IGSF3 | 2.4 | **217** | C1orf116 | 2.1 |
| **58** | BMPER | 3.3 | **138** | OR6C76 | 2.4 | **218** | TMPRSS11B | 2.1 |
| **59** | CTTNBP2 | 3.3 | **139** | ARID5B | 2.4 | **219** | MYH9 | 2.1 |
| **60** | ENC1 | 3.3 | **140** | ANGPT2 | 2.4 | **220** | ME3 | 2.1 |
| **61** | LOC100507003 | 3.2 | **141** | MFGE8* | 2.4 | **221** | PERP* | 2.1 |
| **62** | RGS1* | 3.2 | **142** | LRRC32 | 2.4 | **222** | MC4R | 2.1 |
| **63** | FHOD3 | 3.2 | **143** | IL6ST | 2.4 | **223** | LTBP1 | 2.1 |
| **64** | CREB3L3 | 3.2 | **144** | TP53INP1* | 2.4 | **224** | RTN1 | 2 |
| **65** | ITGA11* | 3.2 | **145** | FRY | 2.4 | **225** | GPR183 | 2 |
| **66** | SERPINI1 | 3.1 | **146** | KRT79 | 2.4 | **226** | RHOB* | 2 |
| **67** | C15orf26 | 3.1 | **147** | ZMAT3 | 2.4 | **227** | STX1A | 2 |
| **68** | AQP1 | 3.1 | **148** | NRP2* | 2.3 | **228** | P2RY14 | 2 |
| **69** | OLFM2 | 3.1 | **149** | CPEB4 | 2.3 | **229** | LOC221272 | 2 |
| **70** | RUNX2 | 3.1 | **150** | FOLR1 | 2.3 | **230** | SLFN5 | 2 |
| **71** | PHOSPHO1 | 3.1 | **151** | NEB | 2.3 | **231** | HIC1 | 2 |
| **72** | HDAC5 | 3 | **152** | PPAPDC1A | 2.3 | **232** | STX11 | 2 |
| **73** | SYNJ2 | 3 | **153** | SPRY4 | 2.3 | **233** | GOLGA6L10 | 2 |
| **74** | ABHD2 | 3 | **154** | ASIC3 | 2.3 | **234** | HPGD | 2 |
| **75** | MAGI3 | 3 | **155** | LTBP3 | 2.3 | **235** | ATP8B4 | 2 |
| **76** | ALAS2 | 3 | **156** | MED12L* | 2.3 | **236** | GOLGA6L10 | 2 |
| **77** | PAPSS2 | 3 | **157** | LINC00642 | 2.3 | **237** | PROS1 | 2 |
| **78** | LMO7 | 3 | **158** | LOC285972 | 2.3 | **238** | MAGED2 | 2 |
| **79** | CD3G | 2.9 | **159** | C3 | 2.3 | **239** | SFRP4* | 2 |
| **80** | CALCOCO1 | 2.9 | **160** | GOLGA6L4 | 2.3 | **240** | GOLGA6L10 | 2 |
