# Supplementary material for: A molecular signature of dormancy in CD34+CD38- acute myeloid leukaemia cells
Source: Oncotarget. 2017 Nov 30;8(67):111405–18. doi: 10.18632/oncotarget.22808 (PMC5762331; doi:10.18632/oncotarget.22808)
Supplement: Supplementary file 3 [file oncotarget-08-111405-s003.docx]

**Supplementary table S2:** The 136 significantly downregulated genes in dormant TF1-a cells

Genes marked with an asterisk are grouped in Figure 3:-

Adhesion related *

Stemness and differentiation-related *

Tumour-suppressor and proliferation regulator genes *

|  | **Gene Symbol** | **Fold-Change** |  | **Gene Symbol** | **Fold-Change** |  | **Gene Symbol** | **Fold-Change** |
| --- | --- | --- | --- | --- | --- | --- | --- | --- |
| **1** | TARP | -11.5 | **47** | FJX1 | -2.6 | **93** | CA2 | -2.2 |
| **2** | UBASH3A | -4.8 | **48** | FAM89A | -2.6 | **94** | SETD7 | -2.2 |
| **3** | RNASE1* | -4.7 | **49** | ECHDC3 | -2.6 | **95** | TNFRSF8* | -2.2 |
| **4** | BNIP3 | -4.6 | **50** | IFITM4P | -2.6 | **96** | DHRS13 | -2.2 |
| **5** | FREM1 | -4.2 | **51** | MT2A | -2.6 | **97** | GNG12 | -2.2 |
| **6** | PCSK9 | -3.9 | **52** | LOC100506025 | -2.6 | **98** | PPP1R14C | -2.2 |
| **7** | FUT1 | -3.9 | **53** | OTTHUMG00000002203 | -2.6 | **99** | HIST1H3F | -2.2 |
| **8** | SH2D1B | -3.8 | **54** | MIR3676 | -2.5 | **100** | GDF3** | -2.2 |
| **9** | CRISP3 | -3.7 | **55** | ACPP | -2.5 | **101** | OSBPL6 | -2.2 |
| **10** | ALDH1A1* | -3.6 | **56** | NFIB* | -2.5 | **102** | EPHA3 | -2.2 |
| **11** | MS4A3 | -3.6 | **57** | CSF2RB* | -2.5 | **103** | ZDHHC19 | -2.2 |
| **12** | CNRIP1 | -3.5 | **58** | SCGN | -2.5 | **104** | SLC25A43 | -2.1 |
| **13** | CEBPA** | -3.5 | **59** | IFITM3* | -2.5 | **105** | PRTG | -2.1 |
| **14** | STAR | -3.5 | **60** | GALM* | -2.5 | **106** | HOXB9 | -2.1 |
| **15** | BST2 | -3.5 | **61** | LGALSL | -2.5 | **107** | VPS37C | -2.1 |
| **16** | PRG2 | -3.5 | **62** | NMU | -2.5 | **108** | BCAT2 | -2.1 |
| **17** | RAB39A | -3.4 | **63** | NRN1 | -2.4 | **109** | MYO16* | -2.1 |
| **18** | EPB42 | -3.3 | **64** | ANKRD27 | -2.4 | **110** | INSIG1 | -2.1 |
| **19** | RHD | -3.3 | **65** | LOC100506636 | -2.4 | **111** | SCRT2 | -2.1 |
| **20** | MS4A4A | -3.3 | **66** | LINC00884 | -2.4 | **112** | SCMH1 | -2.1 |
| **21** | LINC00534 | -3.2 | **67** | MVK | -2.4 | **113** | VEGFA* | -2.1 |
| **22** | FAM86JP | -3.1 | **68** | SNAI1 | -2.4 | **114** | TFR2 | -2.1 |
| **23** | CA14 | -3.1 | **69** | NTN1* | -2.4 | **115** | APLP1 | -2.1 |
| **24** | HBBP1 | -3.0 | **70** | LRIG3 | -2.4 | **116** | LINC00282 | -2.1 |
| **25** | LOC730101 | -3.0 | **71** | HPN-AS1 | -2.4 | **117** | OTTHUMG00000170068 | -2.1 |
| **26** | CD24* | -3.0 | **72** | CECR5 | -2.4 | **118** | PLK2 | -2.1 |
| **27** | SMAGP | -3.0 | **73** | PLD6 | -2.4 | **119** | EDARADD | -2.1 |
| **28** | OTTHUMG00000161282 | -2.9 | **74** | TEX15 | -2.4 | **120** | RHPN1-AS1 | -2.1 |
| **29** | TNFRSF1B* | -2.9 | **75** | GPX8 | -2.3 | **121** | TYSND1 | -2.1 |
| **30** | FEV | -2.9 | **76** | TRIB3 | -2.3 | **122** | UBTD2 | -2.1 |
| **31** | TIMP3 | -2.9 | **77** | TRIM6-TRIM34 | -2.3 | **123** | KCTD3 | -2.1 |
| **32** | BCRP3 | -2.9 | **78** | CALB1 | -2.3 | **124** | ADAMTS1* | -2.1 |
| **33** | SOSTDC1 | -2.8 | **79** | LOC100132147 | -2.3 | **125** | SPATC1L | -2.1 |
| **34** | PYGL | -2.8 | **80** | LINC00341 | -2.3 | **126** | RPL13P5 | -2.1 |
| **35** | MARCKS | -2.8 | **81** | CTSL2 | -2.3 | **127** | GPR85 | -2.1 |
| **36** | CA8 | -2.8 | **82** | PLCXD1 | -2.2 | **128** | SLC35F6 | -2.1 |
| **37** | OTTHUMG00000170886 | -2.8 | **83** | NRCAM* | -2.2 | **129** | GSTA2 | -2.1 |
| **38** | DBP | -2.8 | **84** | RP9P | -2.2 | **130** | LSS | -2.1 |
| **39** | HIST2H4B | -2.7 | **85** | ID3 | -2.2 | **131** | APOC1 | -2.1 |
| **40** | PTX3 | -2.7 | **86** | MAN2A2 | -2.2 | **132** | PCBD1 | -2.0 |
| **41** | NQO1 | -2.7 | **87** | DRAM1 | -2.2 | **133** | PKN3 | -2.0 |
| **42** | AHSP | -2.7 | **88** | LOC100506776 | -2.2 | **134** | CTSH | -2.0 |
| **43** | YME1L1 | -2.7 | **89** | PLEKHF1 | -2.2 | **135** | LINC00891 | -2.0 |
| **44** | SCML2 | -2.7 | **90** | CRYM | -2.2 | **136** | PPM1H | -2.0 |
| **45** | CNKSR3 | -2.6 | **91** | HMGCS1 | -2.2 |  |  |  |
| **46** | ARL4D | -2.6 | **92** | CLDN7 | -2.2 |  |  |  |
