# Supplementary material for: A molecular signature of dormancy in CD34+CD38- acute myeloid leukaemia cells
Source: Oncotarget. 2017 Nov 30;8(67):111405–18. doi: 10.18632/oncotarget.22808 (PMC5762331; doi:10.18632/oncotarget.22808)
Supplement: Supplementary file 4 [file oncotarget-08-111405-s004.docx]

**Supplementary table S4:** The significantly enriched biological processes/signalling pathways in the 240 genes upregulated genes in dormant AML cells*.

| Signalling Pathway/Biological process | Enrichment Score | Enrichment p value | Genes in list in group | Genes not in list in group | GO ID |
| --- | --- | --- | --- | --- | --- |
| Integrin-mediated signalling pathway | 14.43 | 0.00 | 8 | 55 | 7229 |
| Cell adhesion | 13.41 | 0.00 | 21 | 536 | 7155 |
| Response to laminar fluid shear stress | 10.48 | 0.00 | 3 | 3 | 34616 |
| Negative regulation of cell migration | 10.03 | 0.00 | 6 | 51 | 30336 |
| Positive regulation of endothelial cell migration | 9.60 | 0.00 | 4 | 16 | 10595 |
| Negative regulation of blood vessel endothelial cell migration | 8.72 | 0.00 | 3 | 7 | 43537 |
| Leukocyte migration | 8.49 | 0.00 | 7 | 99 | 50900 |
| Cellular component organization or biogenesis | 8.41 | 0.00 | 15 | 440 | 7596 |
| Positive regulation of angiogenesis | 8.02 | 0.00 | 5 | 48 | 45766 |
| Negative regulation of low density lipoprotein particles receptor biosynthesis process | 7.87 | 0.00 | 2 | 1 | 45715 |
| Negative regulation of pathway-restricted SMAD protein phosphorylation | 7.87 | 0.00 | 2 | 1 | 60394 |
| Positive regulation of Apoptosis | 7.71 | 0.00 | 8 | 150 | 43065 |
| Negative regulation of transforming growth factor beta receptor signalling pathway | 7.59 | 0.00 | 4 | 29 | 30512 |
| Osteoblast differentiation | 6.75 | 0.00 | 4 | 37 | 1649 |
| Tens fibre cell differentiation | 6.68 | 0.00 | 2 | 6 | 70306 |
| Negative regulation of insulin-like growth factor receptor signalling pathway | 6.68 | 0.00 | 3 | 22 | 30514 |
| Positive regulation of endothelial cell proliferation | 6.48 | 0.00 | 4 | 40 | 1938 |
| Chemotaxis | 6.09 | 0.00 | 6 | 112 | 6953 |
| Negative regulation of BMP signalling pathway | 5.90 | 0.00 | 3 | 22 | 30514 |
| Negative regulation of smooth muscle migration | 5.68 | 0.00 | 2 | 6 | 14912 |
| Regulation of transport | 5.68 | 0.00 | 2 | 6 | 51049 |
| Negative regulation of Apoptosis | 5.50 | 0.00 | 8 | 216 | 43066 |
| Mitotic cell cycle G1/S transition checkpoint | 5.43 | 0.00 | 2 | 7 | 31575 |
| Muscle contraction | 5.40 | 0.03 | 3 | 58 | 1503 |
| Positive regulation of cell proliferation | 5.32 | 0.00 | 10 | 324 | 8264 |
| Positive regulation of cell adhesion | 5.28 | 0.01 | 3 | 28 | 30155 |
| Response to wounding | 5.23 | 0.01 | 4 | 58 | 9611 |
| Positive regulation of vascular endothelial growth factor production | 4.85 | 0.01 | 2 | 10 | 10575 |
| Axon guidance | 4.69 | 0.01 | 9 | 302 | 7411 |
| Regulation of smooth muscle contraction | 4.69 | 0.01 | 2 | 11 | 6940 |
| Aging | 4.67 | 0.01 | 5 | 106 | 7568 |
| Negative regulation of cell proliferation | 4.65 | 0.01 | 9 | 304 | 8285 |
| Positive regulation of osteoblastic differentiation | 4.63 | 0.01 | 3 | 36 | 45669 |
| Positive regulation of protein kinase B signalling cascade | 4.43 | 0.01 | 3 | 39 | 51897 |
| Osteoblast development | 4.41 | 0.01 | 2 | 13 | 2076 |
| Cell cycle arrest | 4.37 | 0.01 | 5 | 117 | 7050 |
| Bmp signalling pathway | 4.30 | 0.01 | 3 | 41 | 30509 |
| Negative regulation of sequence-specific DNA binding transcription factor activity | 4.13 | 0.02 | 3 | 44 | 43433 |
| Response to vitamin D | 4.05 | 0.02 | 2 | 16 | 33280 |
| Positive regulation of endothelial to mesenchymal transition | 4.05 | 0.02 | 2 | 16 | 10718 |
| Multi-cellular organismal development | 4.05 | 0.02 | 18 | 903 | 7275 |
| Protein localization at cell surface | 4.05 | 0.02 | 2 | 16 | 34394 |
| Positive regulation of pathway-restricted SMAD protein phosphorylation | 4.05 | 0.02 | 2 | 16 | 10662 |
| Intracellular signal transduction | 3.88 | 0.02 | 8 | 290 | 35556 |
| Response to electrical stimulus | 3.85 | 0.02 | 2 | 18 | 51602 |
| Tissue regeneration | 3.76 | 0.02 | 2 | 19 | 42246 |
| Negative regulation of DNA replication | 3.65 | 0.02 | 2 | 18 | 8156 |
| Wnt receptor signalling pathway | 3.51 | 0.03 | 4 | 99 | 16055 |
| Transforming growth factor beta receptor signalling pathway | 3.44 | 0.03 | 3 | 58 | 7179 |
| Response to electrical stimulus | 3.36 | 0.03 | 2 | 24 | 9314 |
| Regulation of signal transduction | 3.36 | 0.03 | 2 | 24 | 9966 |
| Positive regulation of bone mineralization | 3.22 | 0.04 | 2 | 26 | 30501 |
| Immune response | 3.18 | 0.04 | 8 | 334 | 6955 |
| Response to hypoxia | 3.17 | 0.04 | 5 | 162 | 1666 |
| G-protein coupled receptor protein signalling pathway | 3.15 | 0.04 | 8 | 336 | 7186 |
| Cytokine mediated-signalling pathway | 2.89 | 0.04 | 5 | 154 | 19221 |

*****Enrichment analysis using Partek Genomic Suite 6.6 software based on the Kyoto Encyclopaedia of Genes and Genomes (KEGG) database. The groups presented in this table were restricted to groups containing a minimum of 2 genes.
