# Supplementary material for: A molecular signature of dormancy in CD34+CD38- acute myeloid leukaemia cells
Source: Oncotarget. 2017 Nov 30;8(67):111405–18. doi: 10.18632/oncotarget.22808 (PMC5762331; doi:10.18632/oncotarget.22808)
Supplement: Supplementary file 5 [file oncotarget-08-111405-s005.docx]

**Supplementary Table S5:** The significantly enriched biological processes/signalling pathways in the 136 downregulated genes in dormant AML cells*.

| Signalling Pathway/Biological process | Enrichment Score | Enrichment p value | Genes in list in group | Genes not in list in group | GO ID |
| --- | --- | --- | --- | --- | --- |
| negative regulation of fatty acid biosynthetic process | 11.41 | 0.00 | 3 | 5 | 45717 |
| cholesterol biosynthetic process | 10.47 | 0.00 | 4 | 26 | 6695 |
| triglyceride metabolic process | 7.65 | 0.00 | 3 | 23 | 6641 |
| autophagic cell death | 7.57 | 0.00 | 2 | 4 | 48102 |
| response to gonadotropin stimulus | 7.57 | 0.00 | 2 | 4 | 34698 |
| kidney development | 7.36 | 0.00 | 4 | 62 | 1822 |
| notochord development | 7.24 | 0.00 | 2 | 5 | 30903 |
| steroid metabolic process | 6.37 | 0.00 | 4 | 82 | 8202 |
| axonal fasciculation | 5.95 | 0.00 | 2 | 11 | 7413 |
| regulation of phosphorylation | 5.66 | 0.00 | 2 | 13 | 42325 |
| isoprenoid biosynthetic process | 5.66 | 0.00 | 2 | 13 | 8299 |
| cholesterol metabolic process | 5.41 | 0.00 | 3 | 53 | 8203 |
| response to estrogen stimulus | 5.22 | 0.01 | 3 | 57 | 43627 |
| cellular response to starvation | 5.19 | 0.01 | 2 | 17 | 9267 |
| liver development | 4.95 | 0.01 | 3 | 63 | 1889 |
| brown fat cell differentiation | 4.90 | 0.01 | 2 | 20 | 50873 |
| tumour necrosis factor-mediated signalling pathway | 4.81 | 0.01 | 2 | 21 | 33209 |
| cell-cell adhesion | 4.75 | 0.01 | 3 | 68 | 16337 |
| negative regulation of catalytic activity | 4.65 | 0.01 | 2 | 23 | 43086 |
| positive regulation of MAP kinase activity | 4.65 | 0.01 | 2 | 23 | 43406 |
| one-carbon metabolic process | 4.65 | 0.01 | 2 | 23 | 6730 |
| cellular response to organic cyclic compound | 4.57 | 0.01 | 2 | 24 | 71407 |
| negative regulation of fat cell differentiation | 4.57 | 0.01 | 2 | 24 | 45599 |
| steroid biosynthetic process | 4.43 | 0.01 | 2 | 26 | 6694 |
| cell maturation | 4.36 | 0.01 | 2 | 27 | 48469 |
| mesoderm development | 4.36 | 0.01 | 2 | 27 | 7498 |
| lactation | 4.30 | 0.01 | 2 | 28 | 7595 |
| proteolysis | 4.17 | 0.02 | 7 | 431 | 6508 |
| negative regulation of transcription from RNA polymerase II promoter | 4.16 | 0.02 | 6 | 333 | 122 |
| cellular response to hypoxia | 4.11 | 0.02 | 2 | 31 | 71456 |
| positive regulation of neuron apoptosis | 4.11 | 0.02 | 2 | 31 | 43525 |
| growth | 4.11 | 0.02 | 2 | 31 | 40007 |
| lipoprotein metabolic process | 3.90 | 0.02 | 2 | 35 | 42157 |
| central nervous system development | 3.73 | 0.02 | 3 | 101 | 7417 |
| apoptosis | 3.72 | 0.02 | 8 | 583 | 6915 |
| defence response to virus | 3.66 | 0.03 | 2 | 40 | 51607 |
| epithelial cell differentiation | 3.66 | 0.03 | 2 | 40 | 30855 |
| induction of apoptosis by intracellular signals | 3.62 | 0.03 | 2 | 41 | 8629 |
| odontogenesis of dentine-containing tooth | 3.53 | 0.03 | 2 | 43 | 42475 |
| cellular response to mechanical stimulus | 3.38 | 0.03 | 2 | 47 | 71260 |
| cholesterol homeostasis | 3.38 | 0.03 | 2 | 47 | 42632 |
| metabolic process | 3.31 | 0.04 | 4 | 206 | 8152 |
| negative regulation of cell proliferation | 3.25 | 0.04 | 5 | 308 | 8285 |
| cellular response to insulin stimulus | 3.20 | 0.04 | 2 | 52 | 32869 |
| multicellular organismal development | 3.07 | 0.05 | 10 | 911 | 7275 |
| xenobiotic metabolic process | 3.02 | 0.05 | 3 | 135 | 6805 |

*****Enrichment analysis using Partek Genomic Suite 6.6 software based on the Kyoto Encyclopaedia of Genes and Genomes (KEGG) database. The groups presented in this table were restricted to groups containing a minimum of 2 genes.
